# Supplementary material for: Regulating peroxisome–ER contacts via the ACBD5-VAPB tether by FFAT motif phosphorylation and GSK3β
Source: J Cell Biol. 2022 Jan 12;221(3):e202003143. doi: 10.1083/jcb.202003143 (PMC8759595; doi:10.1083/jcb.202003143)
Supplement: Table S1 — lists examples of proteins with a serine/threonine residue at position 5 of the FFAT core. [file JCB_202003143_TableS1.docx]

**Table S1. Examples of proteins with a serine/threonine residue at position 5 of the FFAT core.**

A screen of human proteins with predicted FFAT motifs (score ≤2.5) identified by Slee and Levine (2019) revealed additional proteins, as well as ACBD4 and ACBD5, with a serine/threonine residue at position 5 of the FFAT core. These residues and (predicted) FFAT motifs showed conservation between species. Some of the serine/threonine residues at position 5 have been shown to be phosphorylated (indicated by an asterisk; Hornbeck et al., 2015). The FFAT scores of the shown sequences are indicated (Murphy and Levine, 2016). FFAT motifs with a score of ≤3.5 are highlighted. Light green, acidic tract of the FFAT motif; dark green, FFAT core; orange, serine/threonine residue at position 5 of the FFAT core. Hs, *Homo sapiens* (human); Rn, *Rattus norvegicus* (rat); Mm, *Mus musculus* (mouse); Cl, *Canis lupus familiaris* (dog); Fp, *Falco peregrinus* (falcon); Xt, *Xenopus tropicalis* (frog); Dr, *Danio rerio* (zebrafish).

**ACBD4** - Acyl-CoA-binding domain-containing protein 4 (Q8NC06)

Hs SHSPRDLDSEVFCDSLEQLEPELVWTE **3.5**

Rn SRLPRDLDSEVFCDSVEQLEPELVRVP 3.5

Mm SRPPRDLDSEVFCDSVEQLEPELVRLP 3.5

Cl SQPPRDLDSEVFCDSLEQLEPELVWTE 3.5

Fp SQVTSDSEGDVYCDTLEQMEPEQVSGS 2.5

Xt SLEQLDLDK-DFNDTVTWIYPHLFRPS 3.5

Dr QGPISDTESEVFCDSLEQMDNIKLTAV 2.5

**ACBD5** - Acyl-CoA-binding domain-containing protein 5 (Q5T8D3)

Hs QHLTSDSDSEVYCDSMEQFGQEESLDS **2.5** *

Rn HHLTSDSDSEVYCDSMEQFGQEEYYLG 2.5

Mm HHLTSDSDSEVYCDSMEQFGQEEYYLG 2.5

Cl QHLTSDSDSEVYCDSMEQFGQEESLDS 2.5

Fp QHLTSDSDSEVYCDSMEQLGLEEPLEI 2.5

Xt QHLTSDSDSEIFCDSMEQFGQDEADHS 2.5

Dr HHVASDSDSEVYCDSVDQFGGEDGSEI 2.5

**CALCOCO1** - Calcium-binding and coiled-coil domain-containing protein 1 (Q9P1Z2)

CALCOCO1 has been shown to interact with VAP (Nthiga et al., 2020). Mutations in the FFAT motif strongly reduced the interaction.

Hs DALEDHMDGHFFFSTQDPFTFE 3.0

Rn DALEDHMDGHFFFSTQDPFTFE 3.0

Mm DALEDHMDGHFFFSTQDPFTFE 3.0

Cl DALEDHMDGHFFFSTQDPFTFE 3.0

Fp QGFERHVQTHFDQNV---LNFD 8.5

Xt ---------------------- n/a

Dr ---------------------- n/a

**SNX2** - Sorting nexin-2 (O60749)

SNX2 has been shown to interact with VAPB via two FFAT motifs (Dong et al., 2016). Mutating the phenylalanine (F2) in the FFAT motif below resulted in abolished VAPB binding. Mutating the residue in the other FFAT motif strongly reduced the interaction (DDREDLFAEATEEV – 2.0).

Hs DFEDLEDGEDLFTSTVSTLESSPSSPE **3.5**

Rn DFEELEDGEDLFTSTVSTLESSPSSPE 3.5

Mm DFEELEDGEDLFTSTVSTLESSPSSPE 3.5

Cl DFAELEDGQDLFTSTVSTLESSPSSPE 4.0

Fp --------------------------- n/a

Xt ECEDLEDGEDLFTSTVSTLESSPSSPE 3.5

Dr EQEDSEAAEELFVSVM-------ESPE 5.0

**AKAP11/AKAP220** – A-kinase anchor protein 11 (Q9UKA4)

A peptide containing the FFAT region of the human AKAP11 protein has been shown to bind Scs2p, the major VAP homolog in yeast (Mikitova and Levine, 2012).

Hs DIEDSDSEVSEFFDSFDQFDELEQTLE **1.0**

Rn DIEDSDSEVSEFFDSFDQFDELEQTLE 1.0

Mm DVEDSDSEVSEFFDSFDQFDELEQTLE 1.0

Cl DIEDSDSEVSEFFDSFDQFDELEQTLE 1.0

Fp GIEDSDSEVSEFFDSFDQFDELEQALE 1.0

Xt DVEDSDSELSEFFDSFDQFDETEASLE 1.0

Dr GVEDSDSEVSEFFDSFDQFDELDQSFD 1.0

**JMY** - Junction-mediating and -regulatory protein (Q8N9B5)

Whether the predicted FFAT motif is involved in the JMY-VAP interaction has not been confirmed yet, but JMY has been found to immunoprecipitate with VAPA (Schlüter et al., 2014).

Hs VLFTETDDPEEYYESLSELRQKGYEEV **1.5** *

Rn VLFTETDDPEEYYESLSELRQKGYEEV 1.5

Mm VLFTETDDPEEYYESLSELRQKGYEEV 1.5

Cl VLFTETDDPEEYYESLSELRQKGYEEV 1.5

Fp VLFTETDDPEEYYESLSELRQKGYEEV 1.5

Xt VLFADSDDPEEYYQSLSELRHKGYEEG 3.5

Dr VLFPDSEDAEEYYESLSELRQKGYEDA 1.5

**MYOME** – Myomegalin (Q5VU43)

Hs AGDDTEDTSTEFTDSIEEEAAHHSHQQ **1.5**

Rn AGDETEDTSTQFTDSIEEEAAHNSHQQ 2.5

Mm AGDETEDTSTEFTDSIEEEAAHTSHQQ 1.5

Cl AGEDTEDASTEFTDSIEEEAAHHNHGG 2.0

Fp TGTDADDASSTFTYSIKEEAAHGVATQ 6.0

Xt DNDVDEDSSSQFSDSIEDDTDYQSNGQ 2.5

Dr EEEDEEDCNSEFAGSGEDEKRSKRTAQ 4.0

**ATG2B** - Autophagy-related protein 2 homolog B (Q96BY7)

Hs EESGSEEETLQYFSTVDPNYRSRRKKK **2.0**

Rn EDSGSEEETLQYFSAVDPNYRSRRKKK 1.5

Mm EDSGSEEETLQYFSAVDPNYRSRRKKK 1.5

Cl EESGSEEETLQYFSTVDPNYRSRRKKK 2.0

Fp DESGSEEETLQYYSTVDPNYRSRRRKK 2.5

Xt DDSGSEEETMQHDYIMDSNYHCRRKKK 8.5

Dr --------------------------- n/a

**DYST** - Dystonin (Q03001)

Hs NTGTDT-DSDDDFYDTPLFE-----------DDDHDSLL **2.0**

Rn DTATDS-DSDDYFYDTPLFE-----------DEDHDSLI 3.0

Mm DTATDS-DSDDYFYDTPLFE-----------DEDHDSLI 3.0

Cl VTETDT-DSEGDFYDTPLFE-----------DDDHDSLL 2.0

Fp ---------------KPL--------------------- n/a

Xt LTHTTSSNENYEFYSPEHVVKRACGTSQGHSDNDTSDLE 5.5

Dr STDTEAVRGSQDLYLPSICDIQPEAISTKDKMSHDVSRT 7.0

**PCLO** - Protein piccolo (Q9Y6V0)

Hs RHSWHDED-----DEAFDESPELKYRETKSQE **2.5**

Rn RHSWHDED-----DETFDESPELKFRETKSQE 2.0

Mm RHSWHDED-----DETFDESPELKFRETKSQE 2.0 *

Cl RHSWHDDD-----DDTFDDSPEPRYRETTSQD 2.0

Fp RHSWHDDD-----DDNFDESPEPKYRETKSQD 2.5

Xt RHSWHDDDEEDEDEETYDESPEPKHRETKSQE 2.0

Dr YLSSHDQD-----DQVVQVIPVSEALEV---S 7.7

**CERT** - Ceramide transfer protein (Q9Y5P4)

It has been shown that CERT binds VAP via a FFAT motif (SLINEEEFFDAVEAA – 1.0) (Kawano et al., 2006). CERT has a potential second/non-canonical FFAT motif (Murphy and Levine, 2016). This motif has not been confirmed yet (but is predicted to be in an unstructured region – a characteristic of FFAT motifs).

Hs RDKVVEDDED---------DFPTTRSDGDFLHST-NG **2.5**

Rn RDKVVEDDED---------DFPTTRSDGDFLHNT-NG 2.5

Mm RDKVVEDDED---------DFPTTRSDGDFLHNT-NG 2.5

Cl RDKVVEDDED---------DFPTTRSDGDFLHNT-NG 2.5

Fp RDKGKIVTENSYLRNIQLFTHPTVRSDGDFVHNSNSS 8.0

Xt RDKV-EDDED---------DFLHSHPNGDYIHSS-IG 4.5

Dr RDKV-SDEEE---------DFPTLRPDADYLLNNNNS 4.5
