# Supplementary material for: Regulating peroxisome–ER contacts via the ACBD5-VAPB tether by FFAT motif phosphorylation and GSK3β
Source: J Cell Biol. 2022 Jan 12;221(3):e202003143. doi: 10.1083/jcb.202003143 (PMC8759595; doi:10.1083/jcb.202003143)
Supplement: Table S2 — lists plasmids used in this study. [file JCB_202003143_TableS2.docx]

Table S2. Plasmids used in this study

| Plasmid | Source | Vector |
| --- | --- | --- |
| FLAG-ACBD4.2 | Costello et al., 2017c | pCMV-Tag2B |
| FLAG-ACBD5.1 | Costello et al., 2017b | pCMV-Tag2B |
| FLAG-ACBD5.1 mFFAT | Costello et al., 2017b | pCMV-Tag2B |
| Myc-ACBD5.1 | Costello et al., 2017b | pCMV-Tag3B |
| GSK3β | C. Miller, King’s College, London, UK |  |
| Myc-VAPB | C. Miller, King’s College, London, UK | pCI-neo |
| GST-VAPB | Costello et al., 2017b | pGEX-6p2 |
|  | M. Wilmanns, EMBL, Hamburg, Germany | pETM12 |

Number indicates isoform.
