# Supplementary material for: Regulating peroxisome–ER contacts via the ACBD5-VAPB tether by FFAT motif phosphorylation and GSK3β
Source: J Cell Biol. 2022 Jan 12;221(3):e202003143. doi: 10.1083/jcb.202003143 (PMC8759595; doi:10.1083/jcb.202003143)
Supplement: Table S3 — lists plasmids generated in this study. [file JCB_202003143_TableS3.docx]

Table S3. Plasmids generated in this study

| Plasmid | Template | Primers | Enzymes | Vector |
| --- | --- | --- | --- | --- |
| FLAG-ACBD4.2 mFFAT | FLAG-ACBD4.2 | A4_mFFAT_1_Fw |  | pCMV-Tag2B |
|  |  | A4_mFFAT_1_Rv |  |  |
|  |  | A4_mFFAT_2_Fw |  |  |
|  |  | A4_mFFAT_2_Rv |  |  |
| FLAG-ACBD4.2 with ACBD5 FFAT | Gene synthesis from Eurofins (Table S4) |  | EcoRV | pCMV-Tag2B |
|  |  |  | XhoI |  |
| FLAG-ACBD4.2 S166E | FLAG-ACBD4.2 | A4_S166E_Fw |  | pCMV-Tag2B |
|  |  | A4_S166E_Rv |  |  |
| FLAG-ACBD4.2 S166A | FLAG-ACBD4.2 | A4_S166A_Fw |  | pCMV-Tag2B |
|  |  | A4_S166A_Rv |  |  |
| FLAG -ACBD4.2 S169E | FLAG-ACBD4.2 | A4_S169E_Fw |  | pCMV-Tag2B |
|  |  | A4_S169E_Rv |  |  |
| FLAG-ACBD4.2 S169A | FLAG-ACBD4.2 | A4_S169A_Fw |  | pCMV-Tag2B |
|  |  | A4_S169A_Rv |  |  |
| FLAG-ACBD4.2 S166ES169E | FLAG-ACBD4.2 S166E | A4_(S166E)S169E_Fw |  | pCMV-Tag2B |
|  |  | A4_(S166E)S169E_Rv |  |  |
| FLAG-ACBD4.2 S166AS169A | FLAG-ACBD4.2 S166A | A4_(S166A)S169A_Fw |  | pCMV-Tag2B |
|  |  | A4_(S166A)S169A _Rv |  |  |
| FLAG-ACBD4.2 S166ES169ES171E | FLAG-ACBD4.2 S166ES169E | A4_(S166ES169E)S171E_Fw |  | pCMV-Tag2B |
|  |  | A4_(S166ES169E)S171E_Rv |  |  |
| FLAG-ACBD4.2 S166AS169AS171A | FLAG-ACBD4.2 S166AS169A | A4_(S166AS169A)S171A_Fw |  | pCMV-Tag2B |
|  |  | A4_(S166AS169A)S171A_Rv |  |  |
| FLAG-ACBD4.2 S183E | FLAG-ACBD4.2 | A4_S183E_Fw |  | pCMV-Tag2B |
|  |  | A4_S183E_Rv |  |  |
| FLAG-ACBD4.2 S183A | FLAG-ACBD4.2 | A4_S183A_Fw |  | pCMV-Tag2B |
|  |  | A4_S183A_Rv |  |  |
| FLAG-ACBD5.2 | FLAG-ACBD5.1 | A5_iso2_Fw | EcoRV | pCMV-Tag2B |
|  |  | A5_iso2_Rv | XhoI |  |
| FLAG-ACBD5.2 mFFAT Y266K/C267K/S269R | FLAG-ACBD5.1 mFFAT | A5_iso2_Fw | EcoRV | pCMV-Tag2B |
|  |  | A5_iso2_Rv | XhoI |  |
| FLAG-ACBD5.2 ΔTMD | FLAG-ACBD5.2 | A5_dTMD_S495X_Fw |  | pCMV-Tag2B |
|  |  | A5_dTMD_S495X_Rv |  |  |
| FLAG-ACBD5.2 S259AS261A | FLAG-ACBD5.2 | A5_S259AS261A_Fw |  | pCMV-Tag2B |
|  |  | A5_S259AS261A_Rv |  |  |
| FLAG-ACBD5.2 S259AS261AS263A | FLAG-ACBD5.2 S259AS261A | A5_(S259AS261A)S263A_Fw |  | pCMV-Tag2B |
|  |  | A5_(S259AS261A)S263A_Rv |  |  |
| His-ACBD5 | Gene synthesis from Eurofins (Table S5) |  | NcoI | pETM12 |
|  |  |  | KpnI |  |
| Myc-ACBD5.2 | FLAG-ACBD5.1 | A5_iso2_Fw | EcoRV | pCMV-Tag3B |
|  |  | A5_iso2_Rv | XhoI |  |
| Myc-ACBD5.2 mFFAT Y266K/C267K/S269R | FLAG-ACBD5.1 mFFAT | A5_iso2_Fw | EcoRV | pCMV-Tag3B |
|  |  | A5_iso2_Rv | XhoI |  |
| Myc-ACBD5.2 ΔTMD | Myc-ACBD5.2 | A5_dTMD_S495X_Fw |  |  |
|  |  | A5_dTMD_S495X_Rv |  |  |
| Myc-ACBD5.2 S123AS124A | Myc-ACBD5.2 | A5_S123_124A_Fw |  | pCMV-Tag3B |
|  |  | A5_S123_124A_Rv |  |  |
| Myc-ACBD5.2 S136AT137A | Myc-ACBD5.2 | A5_S136_T137A_Fw |  | pCMV-Tag3B |
|  |  | A5_S136_T137A_Rv |  |  |
| Myc-ACBD5.2 S123AS124AS136AT137A | Myc-ACBD5.2 S123AS124A | A5_S136_T137A_Fw |  | pCMV-Tag3B |
|  |  | A5_S136_T137A_Rv |  |  |
| Myc-ACBD5.2 T252E | Myc-ACBD5.2 | A5_T252E_Fw |  | pCMV-Tag3B |
|  |  | A5_T252E_Rv |  |  |
| Myc-ACBD5.2 T252A | Myc-ACBD5.2 | A5_T252A_Fw |  | pCMV-Tag3B |
|  |  | A5_T252A_Rv |  |  |
| Myc-ACBD5.2 S259E | Myc-ACBD5.2 | A5_S259E_Fw |  | pCMV-Tag3B |
|  |  | A5_S259E_Rv |  |  |
| Myc-ACBD5.2 S259A | Myc-ACBD5.2 | A5_S259A_Fw |  | pCMV-Tag3B |
|  |  | A5_S259A_Rv |  |  |
| Myc-ACBD5.2 S261E | Myc-ACBD5.2 | A5_S261E_Fw |  | pCMV-Tag3B |
|  |  | A5_S261E_Rv |  |  |
| Myc-ACBD5.2 S261A | Myc-ACBD5.2 | A5_S261A_Fw |  | pCMV-Tag3B |
|  |  | A5_S261A_Rv |  |  |
| Myc-ACBD5.2 S263E | Myc-ACBD5.2 | A5_S263E_Fw |  | pCMV-Tag3B |
|  |  | A5_S263E_Rv |  |  |
| Myc-ACBD5.2 S263A | Myc-ACBD5.2 | A5_S263A_Fw |  | pCMV-Tag3B |
|  |  | A5_S263A_Rv |  |  |
| Myc-ACBD5.2 T258AS259A | Myc-ACBD5.2 | A5_T258AS259A_Fw |  | pCMV-Tag3B |
|  |  | A5_T258AS259A_Rv |  |  |
| Myc-ACBD5.2 S259AS261A | Myc-ACBD5.2 | A5_S259AS261A_Fw |  | pCMV-Tag3B |
|  |  | A5_S259AS261A_Rv |  |  |
| Myc-ACBD5.2 S261ES263E | Myc-ACBD5.2 S263E | A5_S261E(S263E)_Fw |  | pCMV-Tag3B |
|  |  | A5_S261E(S263E)_Rv |  |  |
| Myc-ACBD5.2 S261AS263A | Myc-ACBD5.2 S263A | A5_S261A(S263A)_Fw |  | pCMV-Tag3B |
|  |  | A5_S261A(S263A)_Rv |  |  |
| Myc-ACBD5.2 S259ES261ES263E | Myc-ACBD5.2 S261ES263E | A5_S259E(S261ES263E)_Fw |  | pCMV-Tag3B |
|  |  | A5_S259E(S261ES263E)_Rv |  |  |
| Myc-ACBD5.2 S259AS261AS263A | Myc-ACBD5.2 S259AS261A | A5_(S259AS261A)S263A_Fw |  | pCMV-Tag3B |
|  |  | A5_(S259AS261A)S263A_Rv |  |  |
| Myc-ACBD5.2 S269E | Myc-ACBD5.2 | A5_S269E_Fw |  | pCMV-Tag3B |
|  |  | A5_S269E_Rv |  |  |
| Myc-ACBD5.2 S269A | Myc-ACBD5.2 | A5_S269A_Fw |  | pCMV-Tag3B |
|  |  | A5_S269A_Rv |  |  |
| GSK3β S237E ‘mFFAT’ | GSK3β | GSK3b_S237E_Fw |  |  |
|  |  | GSK3b_S237E_Rv |  |  |
| FLAG-VAPB | Myc-VAPB | VAPB_FLAG_Fw | BamHI | pCMV-Tag2B |
|  |  | VAPB_FLAG_Rv | EcoRV |  |
| FLAG-VAPB K87D | FLAG-VAPB | VAPB_K87D_Fw |  | pCMV-Tag2B |
|  |  | VAPB_K87D_Rv |  |  |
| FLAG-VAPB K87DM89D mMSP | FLAG-VAPB K87D | VAPB_(K87D)M89D_Fw |  | pCMV-Tag2B |
|  |  | VAPB_ (K87D)M89D_Rv |  |  |
| Myc-VAPB K87D | Myc-VAPB | VAPB_K87D_Fw |  | pCI-neo |
|  |  | VAPB_K87D_Rv |  |  |
| Myc-VAPB K87DM89D mMSP | Myc-VAPB K87D | VAPB_(K87D)M89D_Fw |  | pCI-neo |
|  |  | VAPB_ (K87D)M89D_Rv |  |  |

Numbering is according to the nucleotide sequence of ACBD4 isoform 2 (UniProt identifier: [Q8NC06-2](https://www.uniprot.org/uniprot/Q8NC06#Q8NC06-2)) and ACBD5 isoform 2 ([Q5T8D3-2](https://www.uniprot.org/uniprot/Q5T8D3#Q5T8D3-2)).
