# Supplementary material for: Regulating peroxisome–ER contacts via the ACBD5-VAPB tether by FFAT motif phosphorylation and GSK3β
Source: J Cell Biol. 2022 Jan 12;221(3):e202003143. doi: 10.1083/jcb.202003143 (PMC8759595; doi:10.1083/jcb.202003143)
Supplement: Table S4 — shows the sequence of ACBD4 with ACBD5 FFAT-like motif region. [file JCB_202003143_TableS4.docx]

Table S4. Sequence of ACBD4 with ACBD5 FFAT-like motif region

| Gene | Source | Sequence (5′ to 3′) |
| --- | --- | --- |
| ACBD4 with ACBD5 FFAT | Eurofins Genomics | GAT ATC ATG GGC ACC GAG AAA GAA AGC CCA GAG CCC GAC TGC CAG AAA CAG TTC CAG GCT GCA GTG AGC GTC ATC CAG AAC CTG CCC AAG AAC GGT TCT TAC CGC CCC TCC TAT GAA GAG ATG CTG CGA TTC TAC AGT TAC TAC AAG CAG GCC ACC ATG GGG CCC TGC CTG GTC CCC CGG CCC GGG TTC TGG GAC CCC ATT GGA CGA TAT AAG TGG GAC GCC TGG AAC AGT CTG GGC AAG ATG AGC AGG GAG GAG GCC ATG TCT GCC TAC ATC ACT GAA ATG AAA CTG GTG GCA CAG AAG GTG ATC GAC ACA GTG CCC CTG GGT GAG GTG GCA GAG GAC ATG TTT GGT TAC TTC GAG CCC CTG TAC CAG GTG ATC CCT GAC ATG CCG AGG CCC CCA GAG ACC TTC CTG AGA AGG GTC ACA GGT TGG AAA GAG CAG GTT GTG AAT GGA GAT GTT GGG GCT GTT TCA GAG CCT CCC TGC CTC CCC AAG GAA CCG GAA GAT GTT ACA GGA ATT CAG CAT TTG ACA AGC GAT TCA GAC AGT GAA GTT TAC TGT GAT TCT ATG GAA CAA TTT GGA CAA GAA GAG TCT TTA GAC AGC TTT ACG GCA GCA TCT GGA GGA AAG CGT GAT CCC AGG AAC AGC CCC GTG CCC CCC ACA AAG AAA GAG GGG TTG CGG GGC AGC CCG CCG GGG CCC CAG GAG TTG GAC GTG TGG CTG CTG GGG ACA GTT CGA GCA CTA CAG GAG AGC ATG CAG GAG GTG CAG GCG AGG GTG CAG AGC CTG GAG AGC ATG CCC CGG CCC CCT GAG CAG AGG CCG CAG CCC AGG CCC AGT GCT CGG CCA TGG CCC CTT GGG CTC CCG GGG CCC GCG CTG CTC TTC TTC CTC CTG TGG CCC TTC GTC GTC CAG TGG CTC TTC CGA ATG TTT CGG ACC CAA AAG AGG TGA CTC GAG |
