# Supplementary material for: Regulating peroxisome–ER contacts via the ACBD5-VAPB tether by FFAT motif phosphorylation and GSK3β
Source: J Cell Biol. 2022 Jan 12;221(3):e202003143. doi: 10.1083/jcb.202003143 (PMC8759595; doi:10.1083/jcb.202003143)
Supplement: Table S5 — shows codon-optimized ACBD5 for expression in E. coli. [file JCB_202003143_TableS5.docx]

Table S5. Codon optimized ACBD5 for expression in *E. coli*

| Gene | Source | Sequence (5′ to 3′) |
| --- | --- | --- |
| ACBD5-codon-optimized | Eurofins Genomics | ATG GCT GAC ACA CGC TCT GTA CAT GAA ACG CGA TTC GAA GCC GCT GTC AAG GTT ATA CAA AGC CTT CCA AAA AAT GGC AGT TTT CAA CCG ACC AAC GAG ATG ATG CTT AAA TTT TAC AGC TTC TAT AAG CAG GCG ACG GAG GGC CCC TGT AAG CTA TCA CGG CCC GGT TTT TGG GAT CCG ATT GGC CGC TAT AAA TGG GAC GCT TGG AGT TCG TTG GGC GAT ATG ACA AAA GAG GAA GCA ATG ATT GCA TAT GTG GAA GAA ATG AAG AAG ATT ATC GAG ACT ATG CCA ATG ACT GAG AAA GTG GAA GAG CTG CTG CGA GTG ATC GGC CCG TTC TAC GAG ATC GTT GAA GAT AAG AAA AGT GGT CGT TCA AGT GAC ATA ACA TCA GAT CTG GGG AAT GTG TTA ACT AGC ACT CCG AAC GCT AAA ACC GTC AAT GGA AAG GCT GAA TCA TCT GAT TCT GGA GCA GAA TCC GAA GAA GAG GAA GCG CAG GAA GAA GTC AAA GGC GCG GAA CAA AGT GAT AAC GAT AAA AAA ATG ATG AAA AAA TCG GCA GAC CAC AAG AAT CTT GAG GTC ATT GTA ACT AAC GGC TAT GAT AAA GAT GGA TTT GTG CAA GAT ATT CAA AAT GAC ATA CAC GCG TCT AGC TCT CTT AAC GGG CGG TCA ACC GAG GAA GTT AAA CCG ATT GAT GAA AAT TTA GGT CAG ACC GGG AAA TCC GCC GTG TGC ATC CAC CAG GAT ATT AAT GAT GAT CAT GTG GAA GAC GTC ACT GGG ATA CAA CAC CTG ACT TCC GAT TCC GAT TCC GAA GTT TAC TGC GAT AGT ATG GAA CAG TTC GGC CAA GAA GAA TCA CTG GAT AGC TTT ACA TCA AAT AAC GGA CCC TTT CAG TAT TAT CTT GGC GGC CAC AGC AGT CAG CCG ATG GAA AAC TCG GGT TTT CGG GAA GAT ATC CAA GTC CCG CCC GGG AAT GGG AAT ATT GGA AAT ATG CAA GTG GTT GCC GTG GAG GGT AAG GGT GAA GTC AAA CAT GGC GGA GAA GAT GGC CGT AAC AAT TCT GGT GCT CCA CAC CGT GAA AAG CGC GGT GGT GAG ACC GAT GAA TTT TCT AAC GTG CGC CGC GGT AGG GGT CAT CGT ATG CAA CAC CTG AGT GAA GGG ACA AAA GGC CGT CAG GTT GGA TCC GGG GGC GAC GGT GAG CGT TGG GGT AGC GAT CGC GGA TCA AGA GGA AGT TTA AAT GAG CAG ATT GCA TTG GTC CTA ATG CGT CTA CAA GAA GAT ATG CAA AAT GTA CTG CAG CGT CTT CAG AAA TTA GAA ACG CTG ACA GCC TTA CAG GCC AAA TCT TCT ACA AGC ACA TTG CAG ACC GCG CCG CAG CCG ACC TCC CAG CGA CCA TCG TGG TGG CCG TTT GAA ATG TGA |
