# Supplementary material for: Regulating peroxisome–ER contacts via the ACBD5-VAPB tether by FFAT motif phosphorylation and GSK3β
Source: J Cell Biol. 2022 Jan 12;221(3):e202003143. doi: 10.1083/jcb.202003143 (PMC8759595; doi:10.1083/jcb.202003143)
Supplement: Table S6 — lists primers used in this study. [file JCB_202003143_TableS6.docx]

Table S6. Primers used in this study

| Name | Sequence (5′ to 3′) |
| --- | --- |
| A4_mFFAT_1_Fw | GACCTGGACTCCGAGGTTGCCTGTGATTCCCTGGAGCAG |
| A4_mFFAT_1_Rv | CTGCTCCAGGGAATCACAGGCAACCTCGGAGTCCAGGTC |
| A4_mFFAT_2_Fw | GACTCCGAGGTTGCCTGTGCTTCCCTGGCGCAGCTGGAGCCTGAGCTG |
| A4_mFFAT_2_Rv | CAGCTCAGGCTCCAGCTGCGCCAGGGAAGCACAGGCAACCTCGGAGTC |
| A4_S166E_Fw | GAACCGGCACCCCCAGAACCAGAGTCCCATTC |
| A4_S166E_Rv | GAATGGGACTCTGGTTCTGGGGGTGCCGGTTC |
| A4_S166A_Fw | GAACCGGCACCCCCAGCCCCAGAGTCCCATTC |
| A4_S166A_Rv | GAATGGGACTCTGGGGCTGGGGGTGCCGGTTC |
| A4_S169E_Fw | CCCCCAAGCCCAGAGGAACATTCACCCAGGGAC |
| A4_S169E_Rv | GTCCCTGGGTGAATGTTCCTCTGGGCTTGGGGG |
| A4_S169A_Fw | CCCCCAAGCCCAGAGGCCCATTCACCCAGGGAC |
| A4_S169A_Rv | GTCCCTGGGTGAATGGGCCTCTGGGCTTGGGGG |
| A4_(S166E)S169E_Fw | CCCCCAGAACCAGAGGAACATTCACCCAGGGAC |
| A4_(S166E)S169E_Rv | GTCCCTGGGTGAATGTTCCTCTGGTTCTGGGGG |
| A4_(S166A)S169A_Fw | CCCCCAGCCCCAGAGGCCCATTCACCCAGGGAC |
| A4_(S166A)S169A_Rv | GTCCCTGGGTGAATGGGCCTCTGGGGCTGGGGG |
| A4_(S166ES169E)S171E_Fw | GAACCAGAGGAACATGAACCCAGGGACCTGGAC |
| A4_(S166ES169E)S171E_Rv | GTCCAGGTCCCTGGGTTCATGTTCCTCTGGTTC |
| A4_(S166AS169A)S171A_Fw | GCCCCAGAGGCCCATGCACCCAGGGACCTGGAC |
| A4_(S166AS169A)S171A_Rv | GTCCAGGTCCCTGGGTGCATGGGCCTCTGGGGC |
| A4_S183E_Fw | GAGGTTTTCTGTGATGAACTGGAGCAGCTGGAG |
| A4_S183E_Rv | CTCCAGCTGCTCCAGTTCATCACAGAAAACCTC |
| A4_S183A_Fw | GAGGTTTTCTGTGATGCCCTGGAGCAGCTGGAG |
| A4_S183A_Rv | CTCCAGCTGCTCCAGGGCATCACAGAAAACCTC |
| A5_iso2_Fw | AAGGCGATATCATGGCGGACACGAGATCCGTG |
| A5_iso2_Rv | GTTCTCGAGTTATCAGTTCAGTTTTCTTCTCCTTCTTTG |
| A5_dTMD_S495X_Fw | TCTTGGTGGCCCTTCGAGATGTGACCTGGTGTGCTAACGTTTGC |
| A5_dTMD_S495X_Rv | GCAAACGTTAGCACACCAGGTCACATCTCGAAGGGCCACCAAGA |
| A5_S123_124A_Fw | CAAAAAGAGTGGCAGGGCTGCTGATATAACCTCAG |
| A5_S123_124A_Rv | CTGAGGTTATATCAGCAGCCCTGCCACTCTTTTTG |
| A5_S136_T137A_Fw | GTAATGTTCTCACTGCTGCTCCAAACGCCAAAACC |
| A5_S136_T137A_Rv | GGTTTTGGCGTTTGGAGCAGCAGTGAGAACATTAC |
| A5_T252E_Fw | CATGTTGAAGATGTTGAAGGAATTCAGCATTTG |
| A5_T252E_Rv | CAAATGCTGAATTCCTTCAACATCTTCAACATG |
| A5_T252A_Fw | CATGTTGAAGATGTTGCAGGAATTCAGCATTTG |
| A5_T252A_Rv | CAAATGCTGAATTCCTGCAACATCTTCAACATG |
| A5_S259E_Fw | CAGCATTTGACAGAGGATTCAGACAGTGAAG |
| A5_S259E_Rv | CTTCACTGTCTGAATCCTCTGTCAAATGCTG |
| A5_S259A_Fw | TTCAGCATTTGACAGCCGATTCAGACAGTGAAGT |
| A5_S259A_Rv | ACTTCACTGTCTGAATCGGCTGTCAAATGCTGAA |
| A5_S261E_Fw | CATTTGACAAGCGATGAAGACAGTGAAGTTTAC |
| A5_S261E_Rv | GTAAACTTCACTGTCTTCATCGCTTGTCAAATG |
| A5_S261A_Fw | CATTTGACAAGCGATGCAGACAGTGAAGTTTAC |
| A5_S261A_Rv | GTAAACTTCACTGTCTGCATCGCTTGTCAAATG |
| A5_S263E_Fw | CAAGCGATTCAGACGAGGAAGTTTACTGTG |
| A5_S263E_Rv | CACAGTAAACTTCCTCGTCTGAATCGCTTG |
| A5_S263A_Fw | CAAGCGATTCAGACGCTGAAGTTTACTGTG |
| A5_S263A_Rv | CACAGTAAACTTCAGCGTCTGAATCGCTTG |
| A5_T258AS259A_Fw | GAATTCAGCATTTGGCAGCCGATTCAGACAGTG |
| A5_T258AS259A_Rv | CACTGTCTGAATCGGCTGCCAAATGCTGAATTC |
| A5_S259AS261A_Fw | TTCAGCATTTGACAGCCGATGCAGACAGTGAAGT |
| A5_S259AS261A_Rv | ACTTCACTGTCTGCATCGGCTGTCAAATGCTGAA |
| A5_S261E(S263E)_Fw | CATTTGACAAGCGATTCAGACGAGGAAGTTTAC |
| A5_S261E(S263E)_Rv | CATTTGACAAGCGATGAAGACGAGGAAGTTTAC |
| A5_S261A(S263A)_Fw | CATTTGACAAGCGATGCAGACGCTGAAGTTTAC |
| A5_S261A(S263A)_Rv | GTAAACTTCAGCGTCTGCATCGCTTGTCAAATG |
| A5_S259E(S261ES263E)_Fw | CAGCATTTGACAGAGGATGAAGACGAGGAAG |
| A5_S259E(S261ES263E)_Rv | CTTCCTCGTCTTCATCCTCTGTCAAATGCTG |
| A5_(S259AS261A)S263A_Fw | CAGCCGATGCAGACGCTGAAGTTTACTGTG |
| A5_(S259AS261A)S263A_Rv | CACAGTAAACTTCAGCGTCTGCATCGGCTG |
| A5_S269E_Fw | GAAGTTTACTGTGATGAAATGGAACAATTTGGAC |
| A5_S269E_Rv | GTCCAAATTGTTCCATTTCATCACAGTAAACTTC |
| A5_S269A_Fw | GAAGTTTACTGTGATGCTATGGAACAATTTGGAC |
| A5_S269A_Rv | GTCCAAATTGTTCCATAGCATCACAGTAAACTTC |
| GSK3b_S237E_Fw | CACTGATTATACCTCTAGTATAGATGTATGGTCTG |
| GSK3b_S237E_Rv | CACTGATTATACCTCTGAGATAGATGTATGGTCTG |
| VAPB_K87D_Fw | GAGAAAAGTAAACACGATTTTATGGTTCAGTC |
| VAPB_K87D_Rv | GACTGAACCATAAAATCGTGTTTACTTTTCTC |
| VAPB_(K87D)M89D_Fw | GTAAACACGATTTTGATGTTCAGTCTATGTTTGC |
| VAPB_(K87D)M89D_Rv | GCAAACATAGACTGAACATCAAAATCGTGTTTAC |
| VAPB_FLAG_Fw | ATAGGATCCATGGCGAAGGTGGAGCAGGTC |
| VAPB_FLAG_Rv | TATGATATCCTACAAGGCAATCTTCCCAA |
