# Supplementary material for: Regulating peroxisome–ER contacts via the ACBD5-VAPB tether by FFAT motif phosphorylation and GSK3β
Source: J Cell Biol. 2022 Jan 12;221(3):e202003143. doi: 10.1083/jcb.202003143 (PMC8759595; doi:10.1083/jcb.202003143)
Supplement: Table S7 — lists primary and secondary antibodies used in this study. [file JCB_202003143_TableS7.docx]

Table S7. Primary and secondary antibodies used in this study

| Antibody | Type | Dilution | | Source |
| --- | --- | --- | --- | --- |
|  |  | WB | IMF |  |
| FLAG | mc ms | 1:2,000 | 1:500 | Sigma-Aldrich F3165 |
| FLAG | pc rb | 1:1,000 |  | Sigma-Aldrich F7425 |
| Myc | mc ms | 1:1,000 | 1:200 | Santa Cruz sc-40 |
| Myc | mc rb | 1:2,000–1:10,000 |  | Abcam ab9106 |
| ACBD5 | pc rb | 1:500 |  | Cambridge Bioscience HPA012145 |
| ACBD5 control | pc rb | 0.5 µg/ml |  | Generated for this study by Eurogentec |
| ACBD5 pS269 | pc rb | 0.5 µg/ml |  | Generated for this study by Eurogentec |
| β-Catenin | pc rb | 1:1000 |  | Cell Signaling 9562 |
| β-Catenin pS33pS37 | mc ms | 1:500 |  | Santa Cruz sc-57535 |
| GSK3β | mc ms | 1:500-1,000 | 1:50 | Santa Cruz sc-377213 |
| GSK3β pS9 | mc ms | 1:500-1,000 |  | Santa Cruz sc-373800 |
| GSK3β pY216 | pc rb | 1:500-1,000 |  | Abcam ab75745 |
| PEX14 | pc rb |  | 1:14,000 | D. Crane, Griffith University, Brisbane, Australia |
| PTPIP51 | pc rb | 1:500 |  | Sigma-Aldrich HPA009975 |
| VAPB | pc rb | 1:500 |  | Abcam ab103638 |
| VAPB | pc rb | 1:1,000 |  | Sigma-Aldrich HPA013144 |
| GAPDH | pc rb | 1:10,000 |  | ProSci 3783 |
| αTubulin | mc ms | 1:1,000 |  | Sigma-Aldrich T9026 |
| HRP IgG | gt anti-rb | 1:10,000 |  | Bio-Rad Laboratories 170-6515 |
| HRP IgG | gt anti-ms | 1:10,000 |  | Bio-Rad Laboratories 170-6516 |
| Alexa Fluor 488 IgG | dk anti-ms |  | 1:400 | Molecular Probes A21202 |
| Alexa Fluor 594 IgG | dk anti-rb |  | 1:1,000 | Molecular Probes A21207 |

Abbreviations: dk, donkey; gt, goat; IMF, immunofluorescence; mc, monoclonal; ms, mouse; pc, polyclonal; rb, rabbit; WB, Western blot.
