# Supplementary figures and images for: Regulating peroxisome–ER contacts via the ACBD5-VAPB tether by FFAT motif phosphorylation and GSK3β
Source: J Cell Biol. 2022 Jan 12;221(3):e202003143. doi: 10.1083/jcb.202003143 (PMC8759595; doi:10.1083/jcb.202003143)

1. A

### FLAG-ACBD4

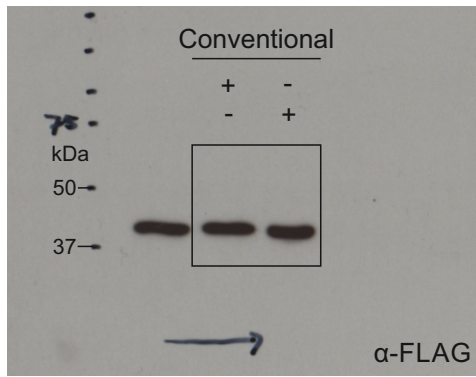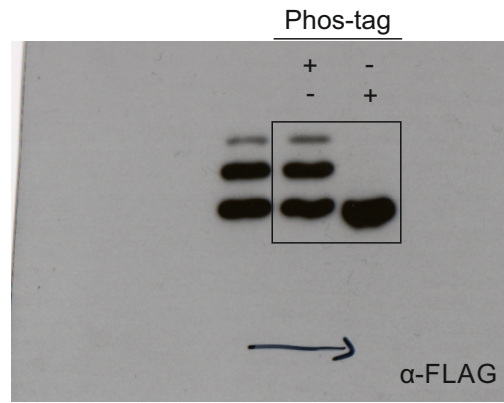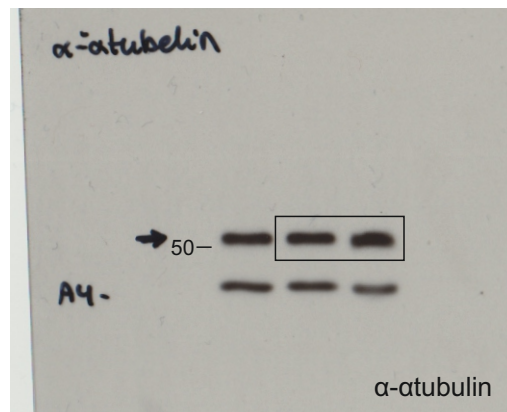

### FLAG-ACBD5

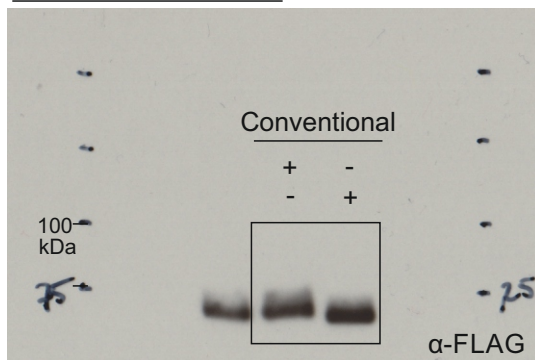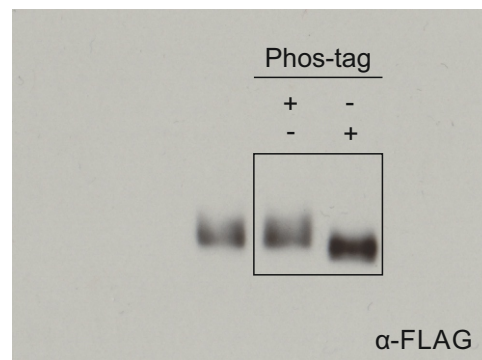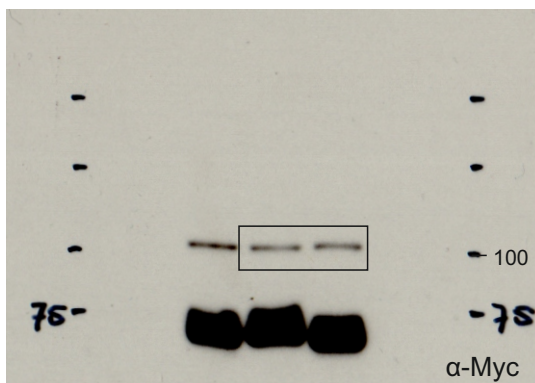

1. B

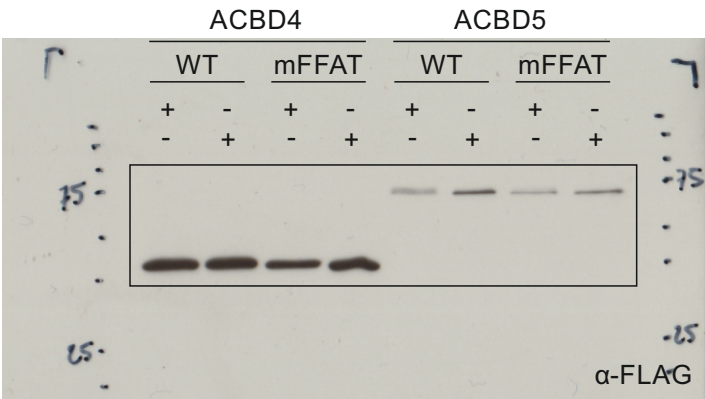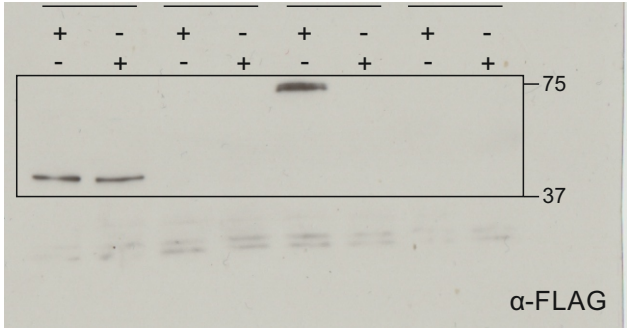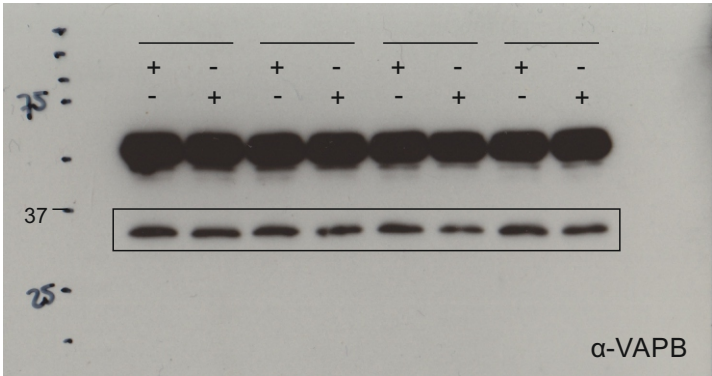

1. C

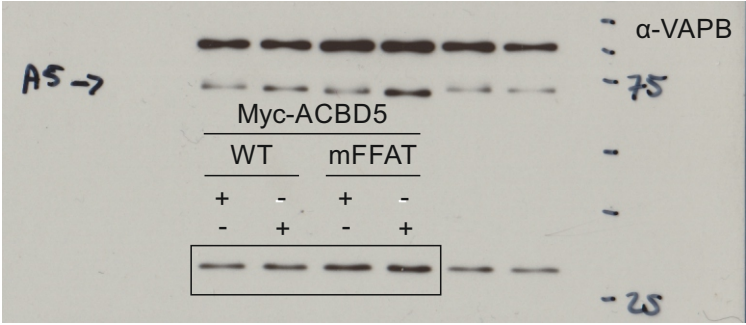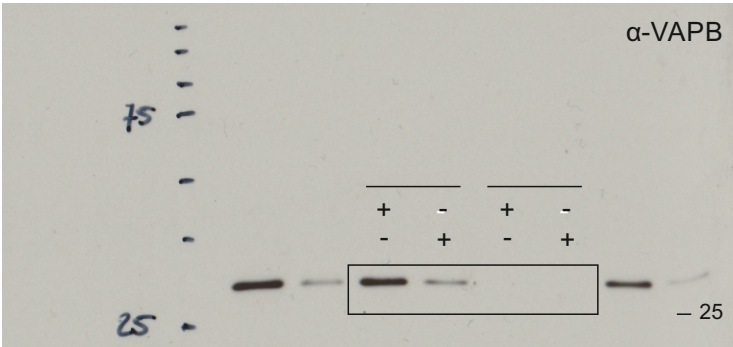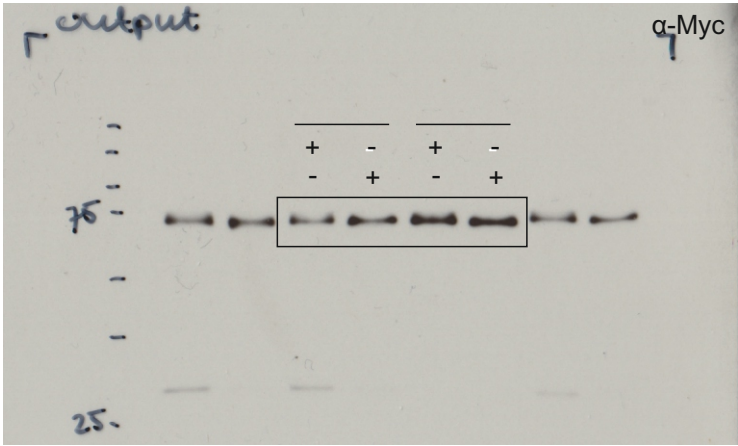

1. D

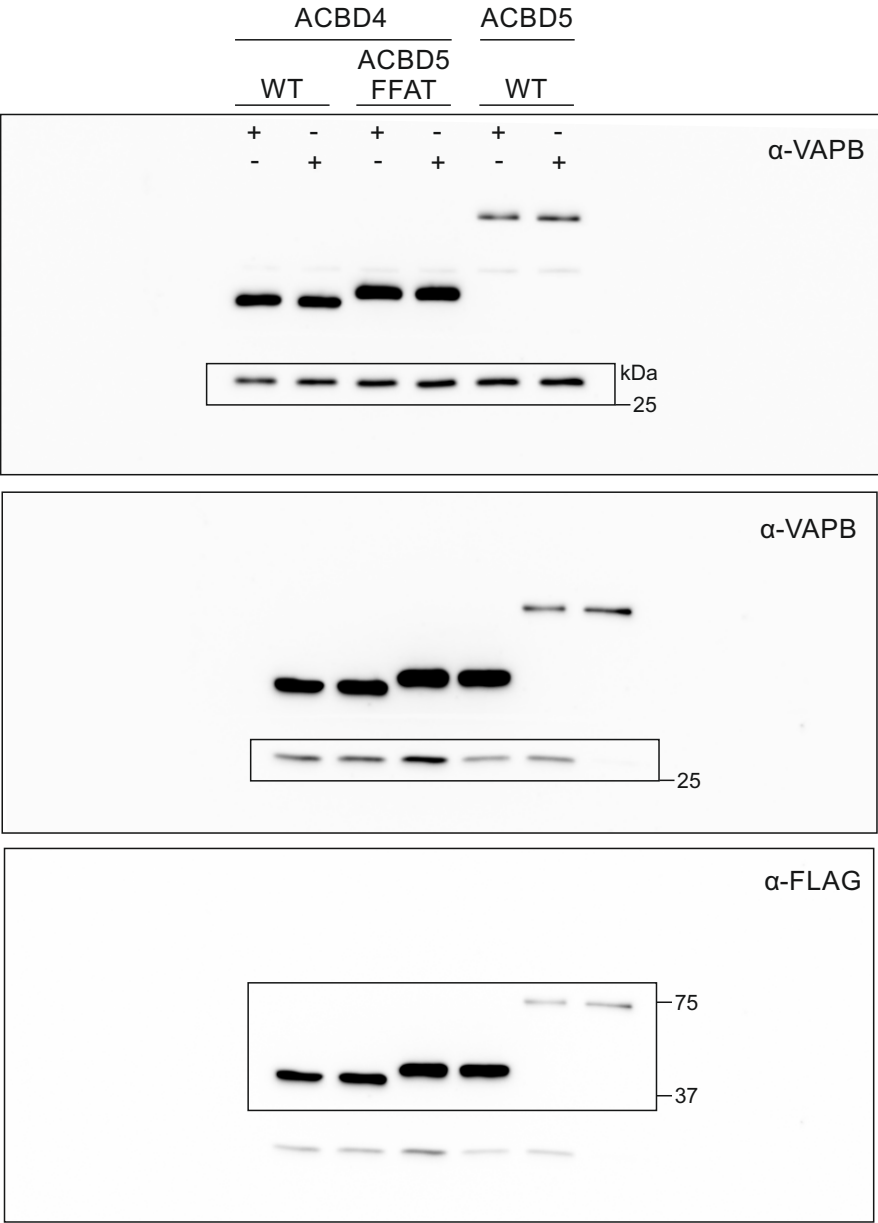

Supplement: SourceData F1 — contains original blots for Fig. 1. [file JCB_202003143_SourceDataF1.pdf]

2. C

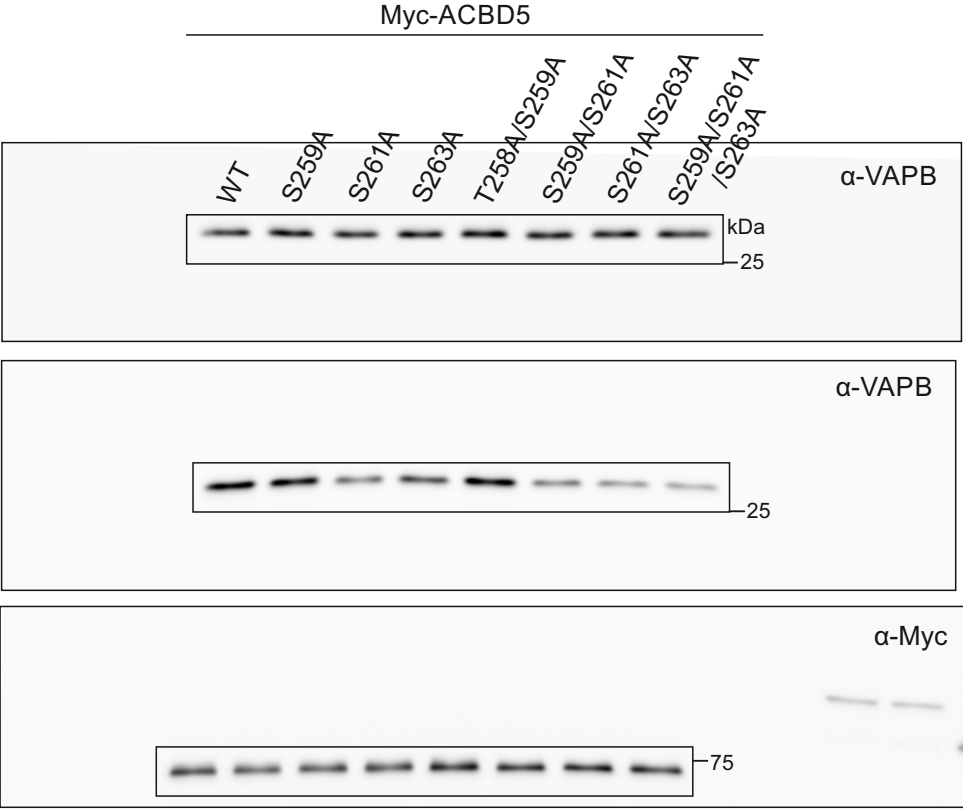

2. D

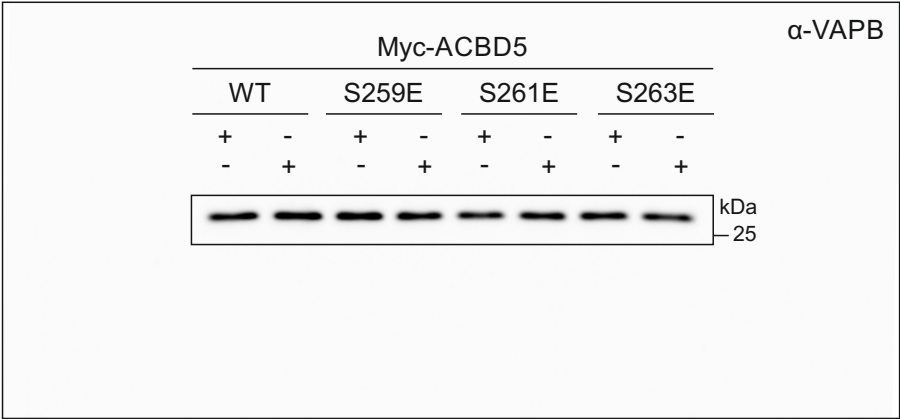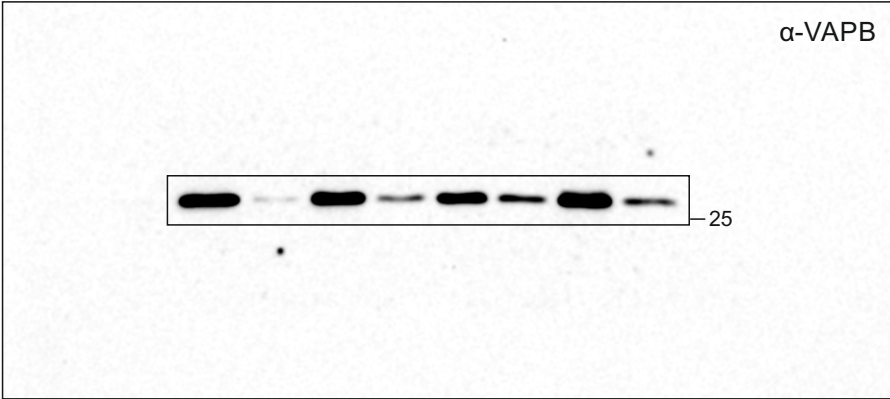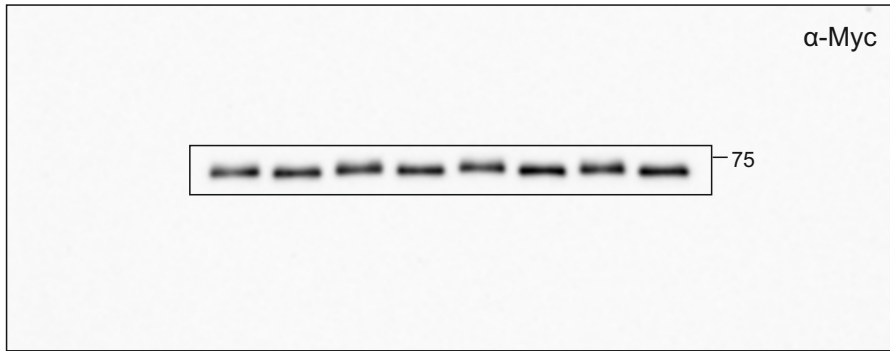

2. E

| Myc-ACBD5 |   |                         |   |
|-----------|---|-------------------------|---|
| WT        |   | S259E<br>S261E<br>S263E |   |
| +         | - | +                       | - |
| -         | + | -                       | + |

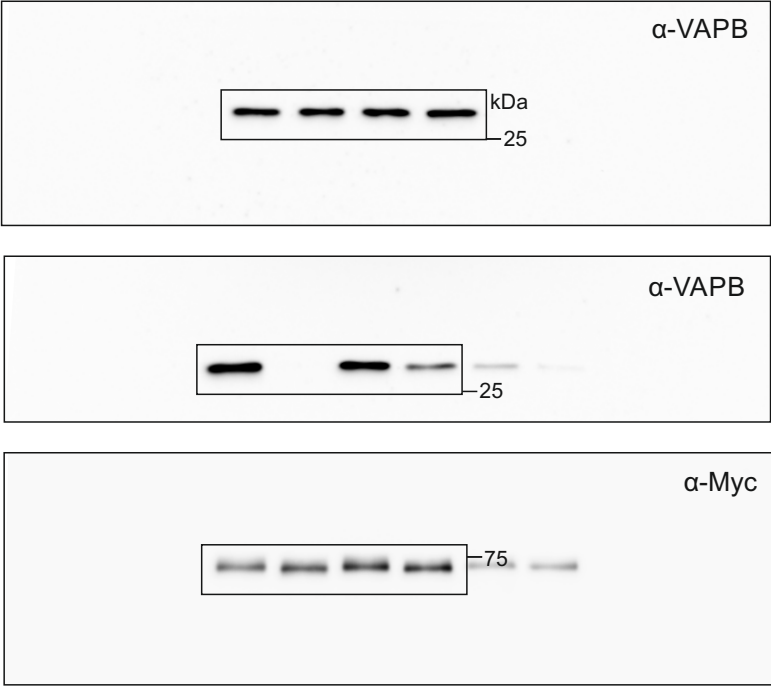

Supplement: SourceData F2 — contains original blots for Fig. 2. [file JCB_202003143_SourceDataF2.pdf]

3. A

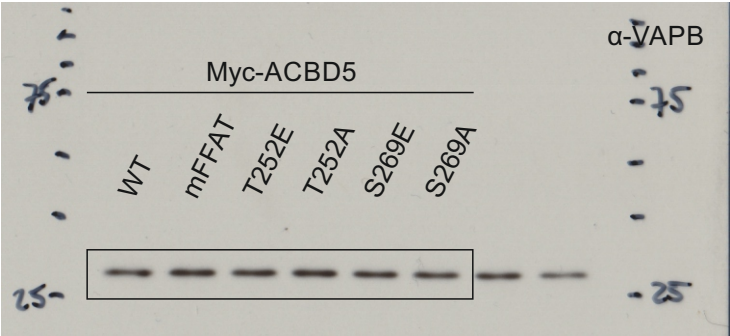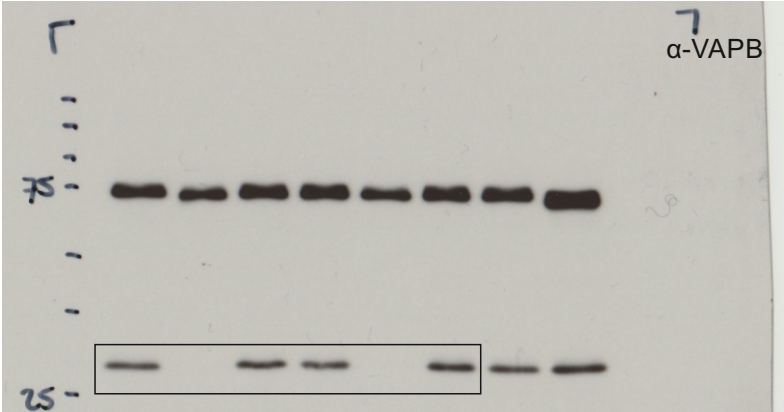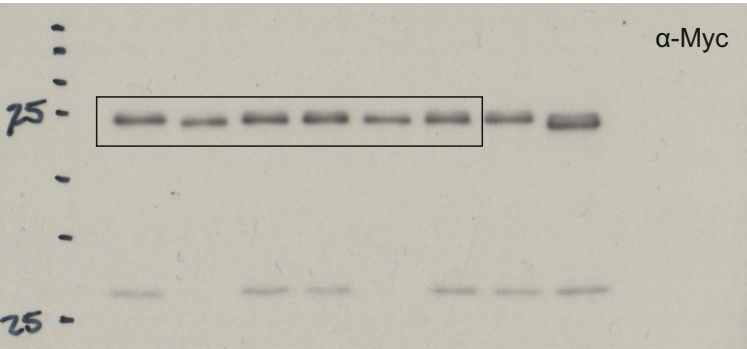

3. C

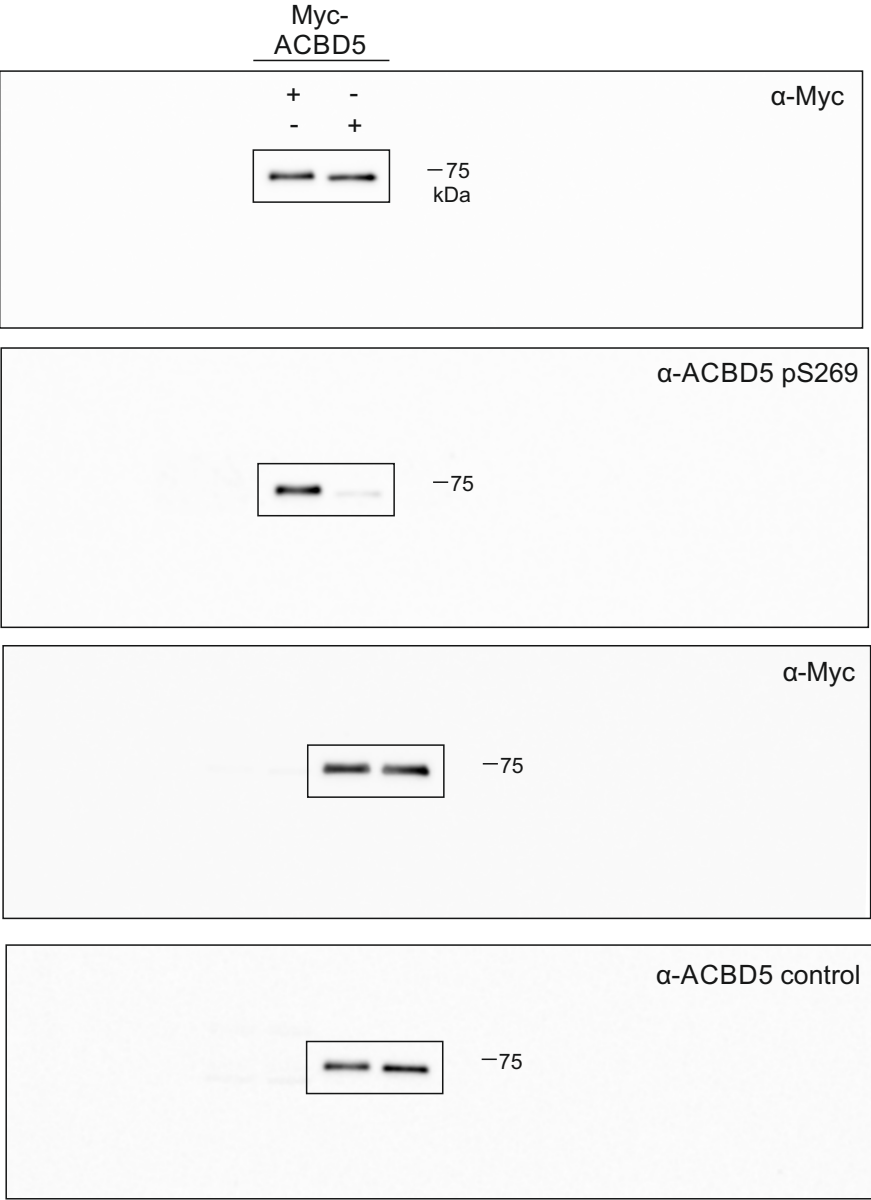

3. D

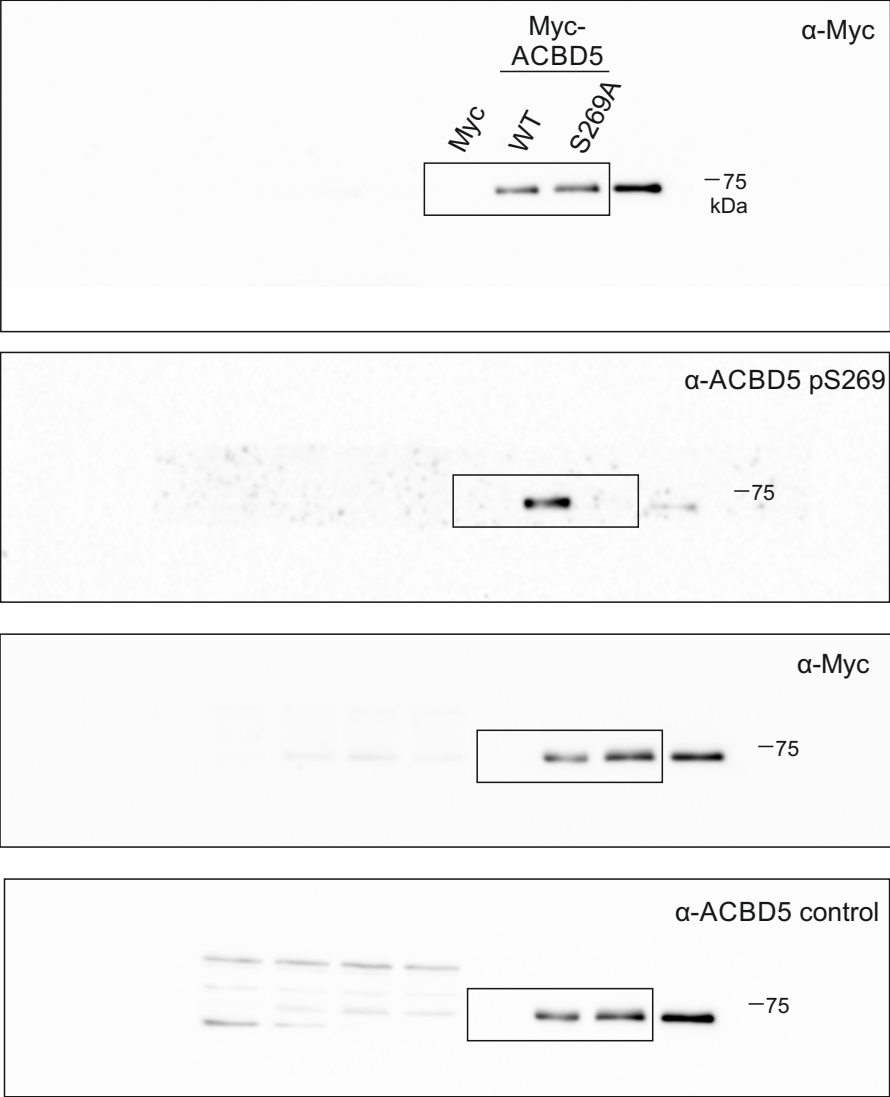

3. E

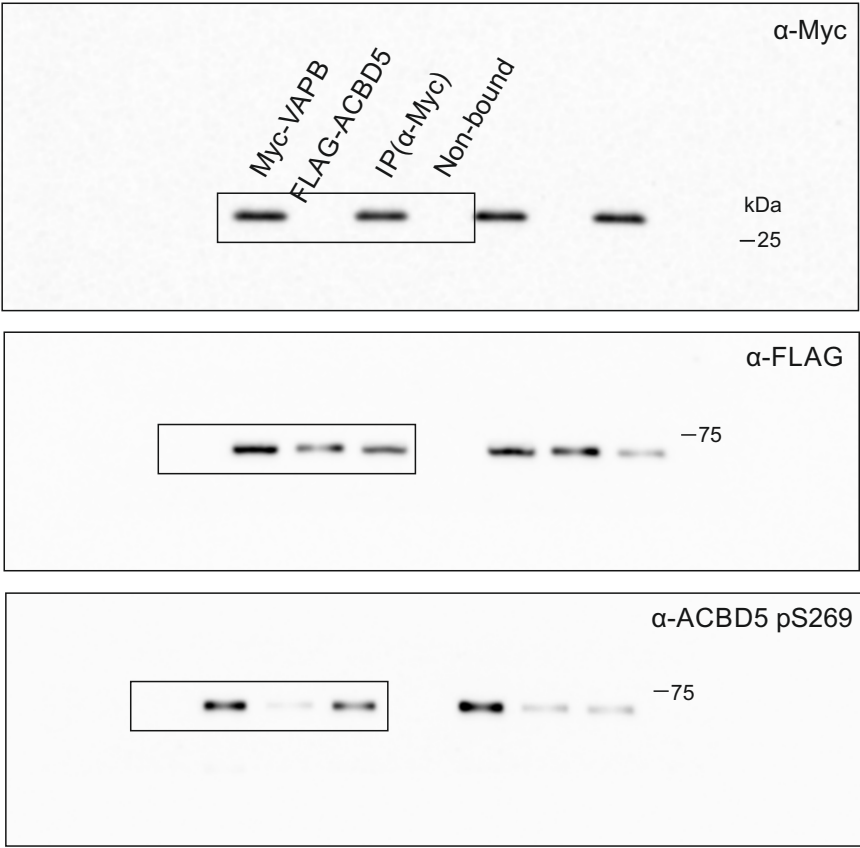

Supplement: SourceData F3 — contains original blots for Fig. 3. [file JCB_202003143_SourceDataF3.pdf]

4. D

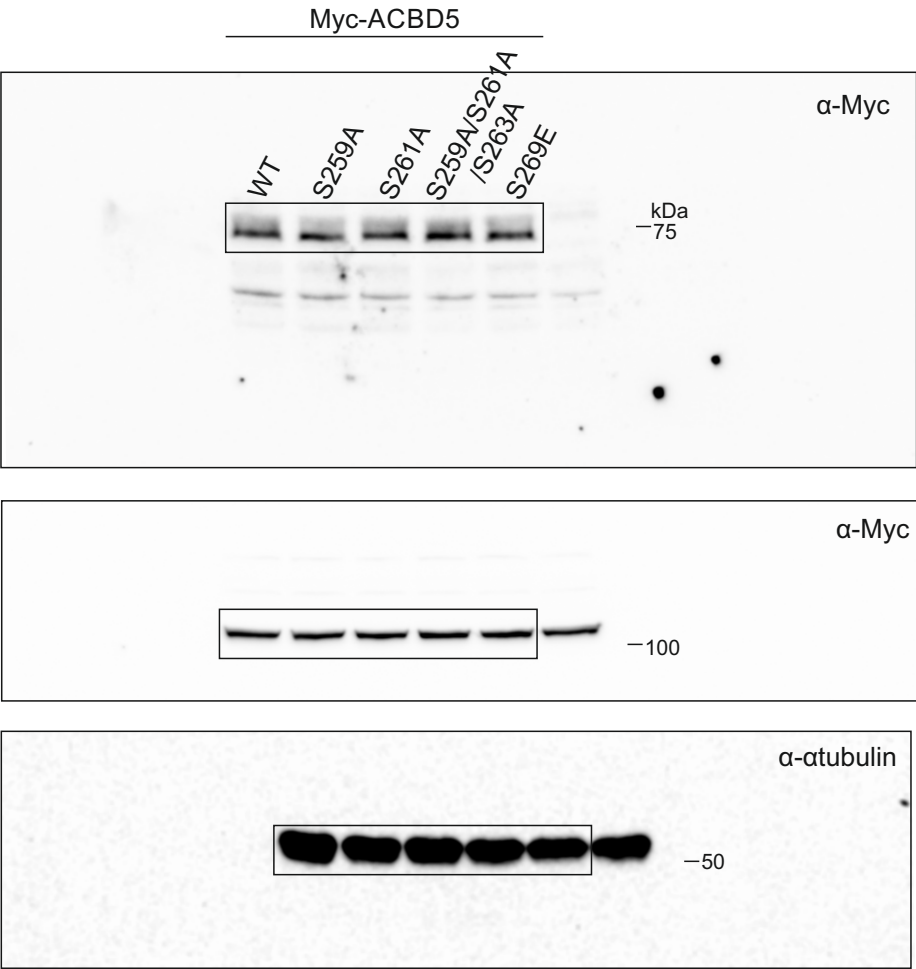

Supplement: SourceData F4 — contains original blots for Fig. 4. [file JCB_202003143_SourceDataF4.pdf]

5. A

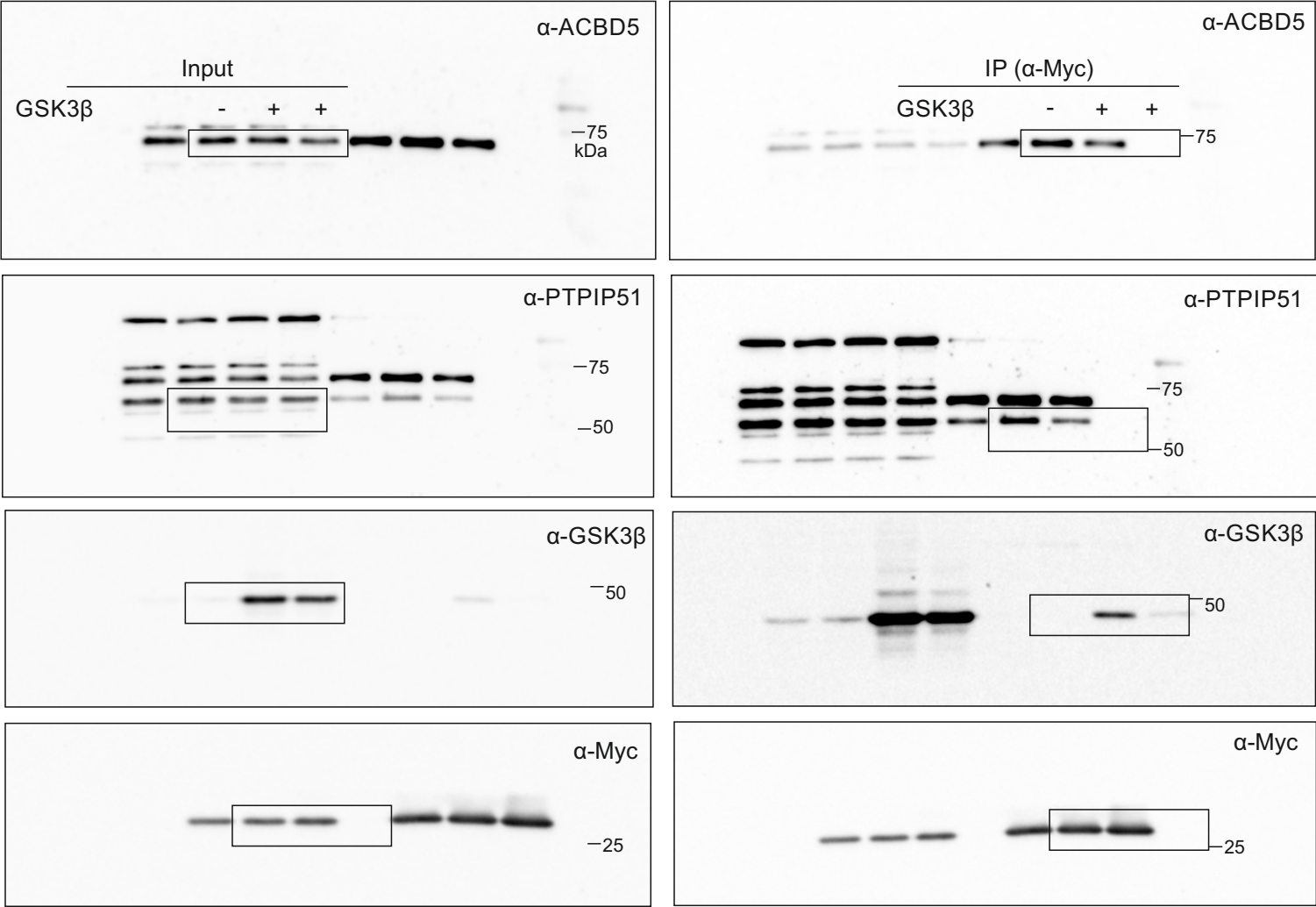

5. B

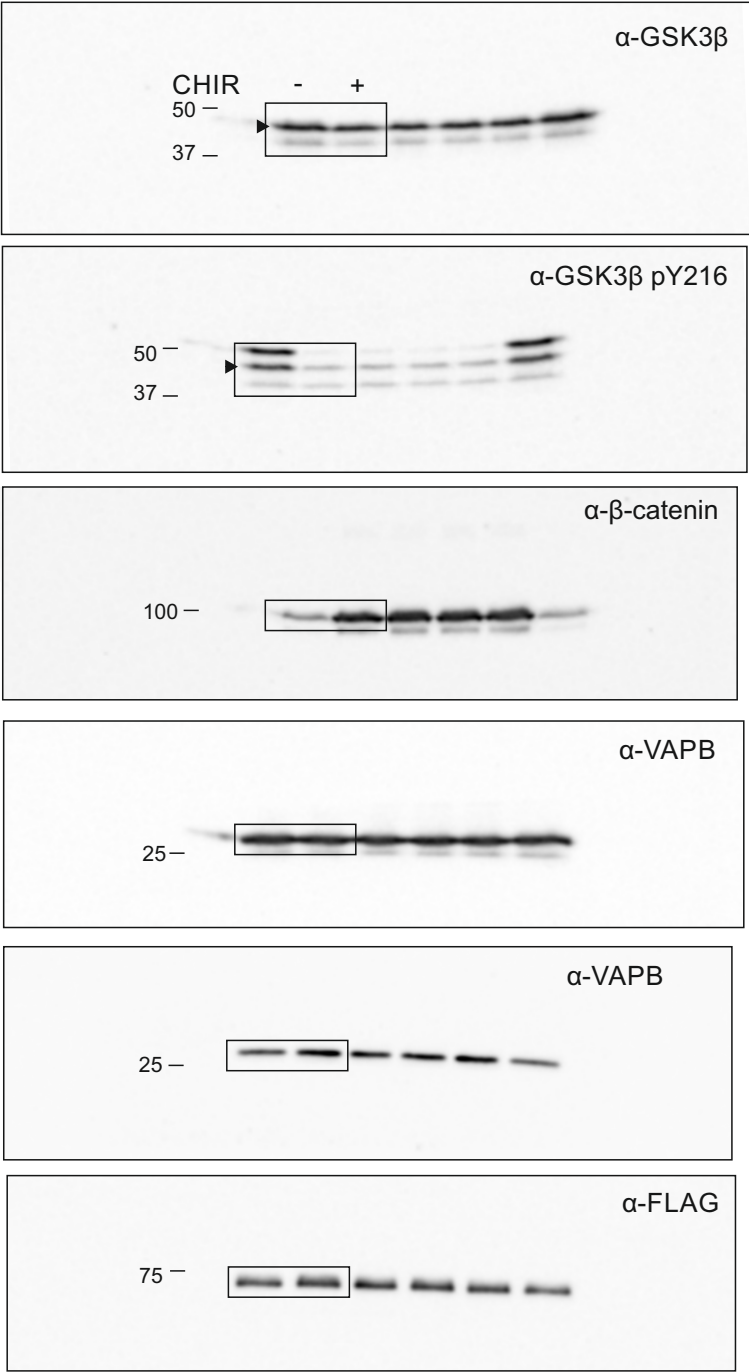

Supplement: SourceData F5 — contains original blots for Fig. 5. [file JCB_202003143_SourceDataF5.pdf]

6. A

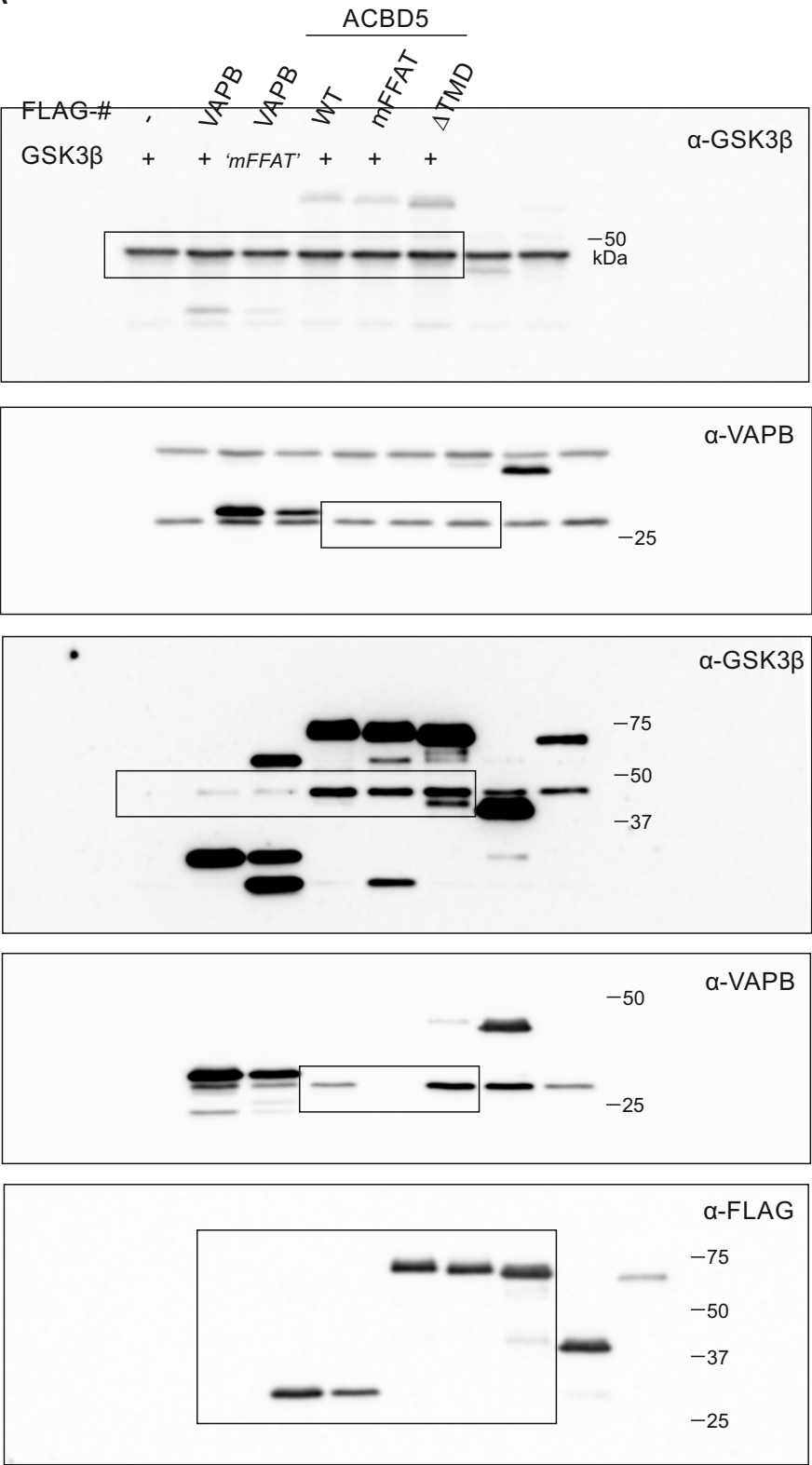

6. B

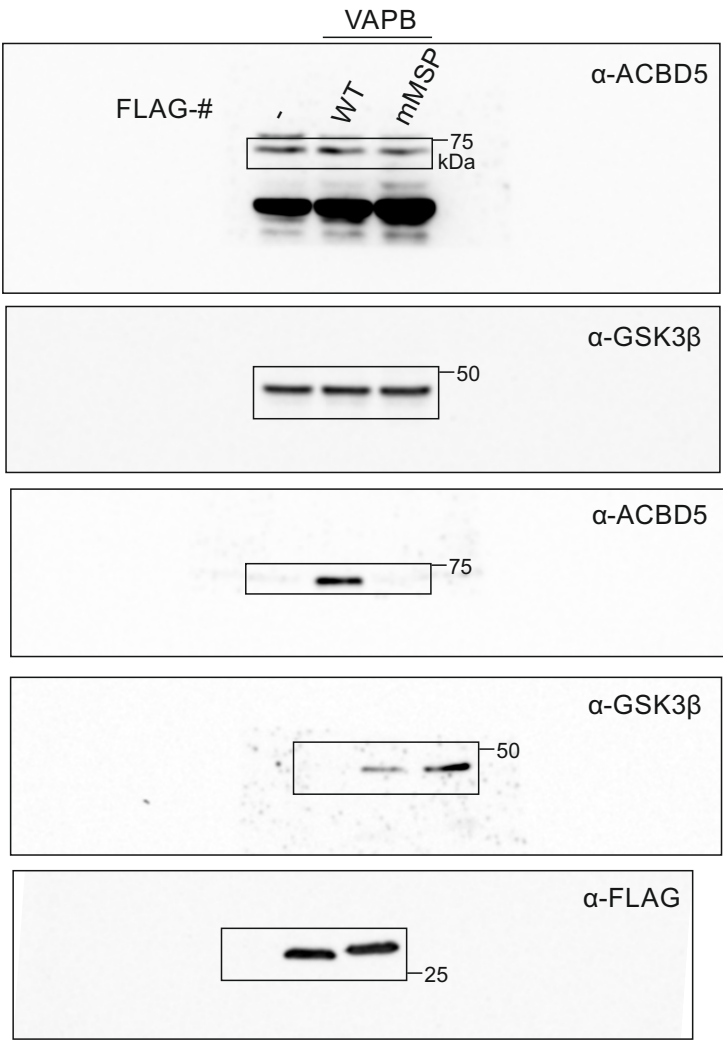

6. C

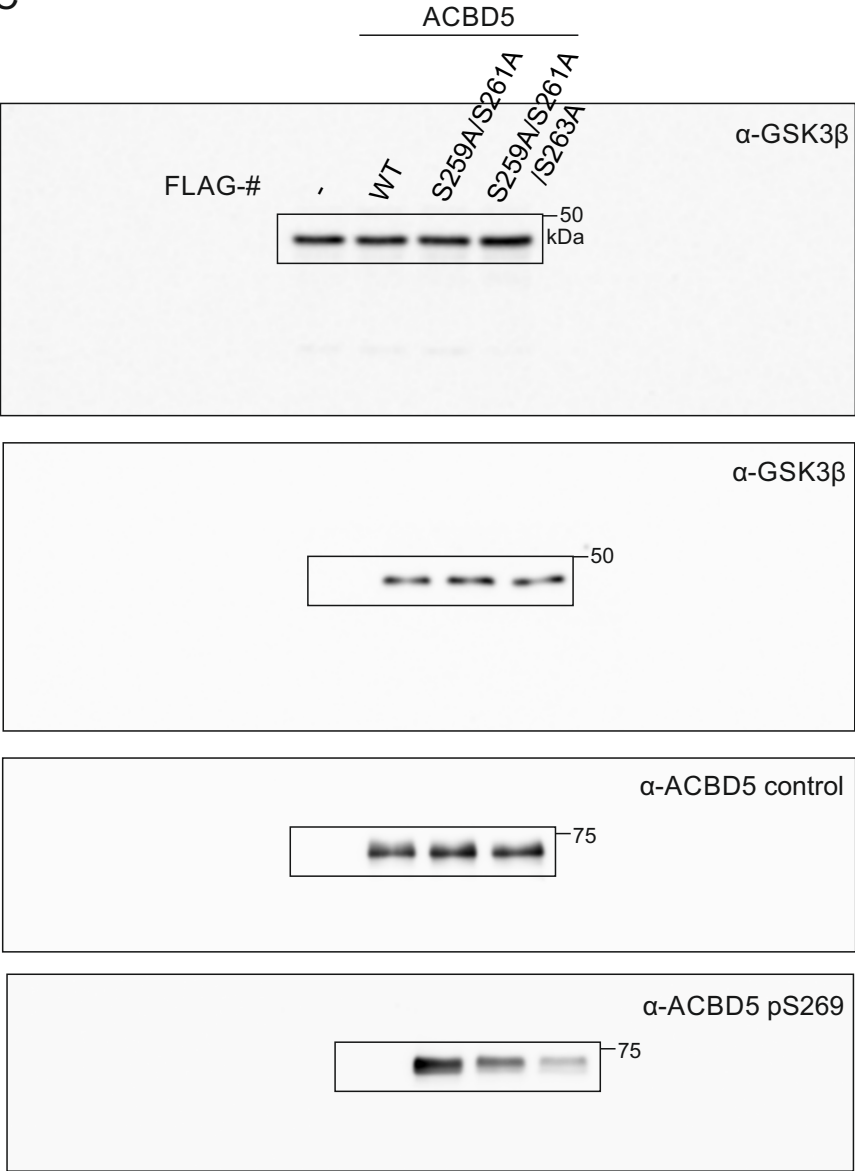

Supplement: SourceData F6 — contains original blots for Fig. 6. [file JCB_202003143_SourceDataF6.pdf]

7. A

Myc-ACBD5      WT   S269A   WT  
GSK3β            K85A   WT     WT

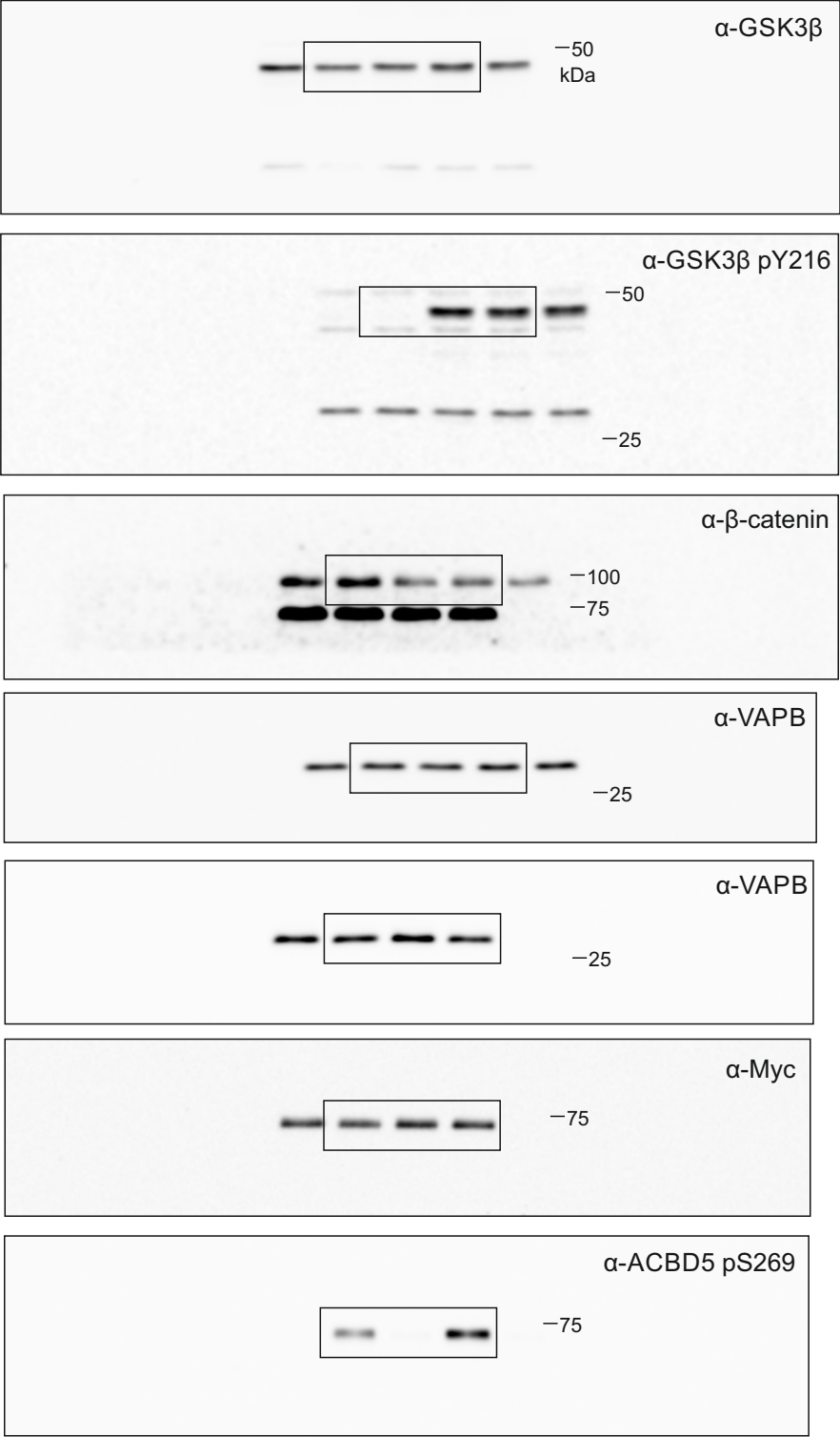

7. B

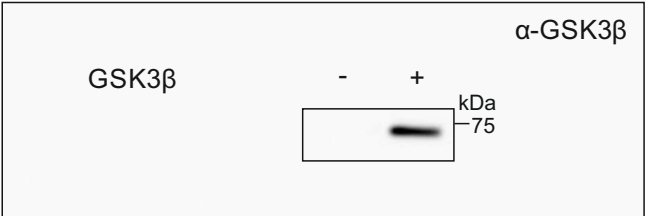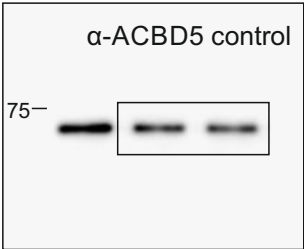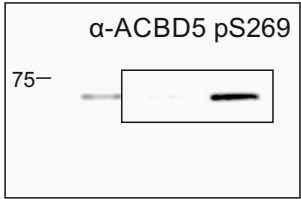

Supplement: SourceData F7 — contains original blots for Fig. 7. [file JCB_202003143_SourceDataF7.pdf]

S1.A

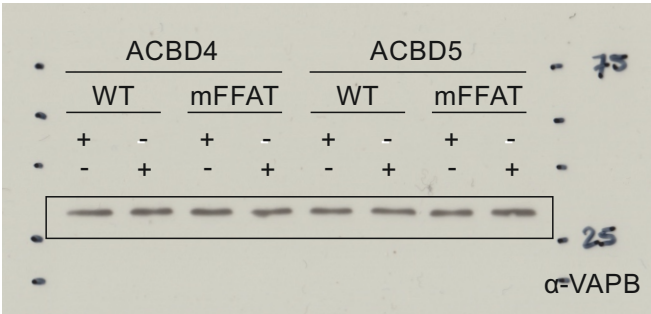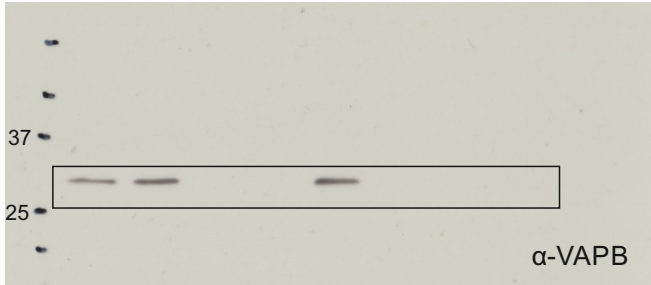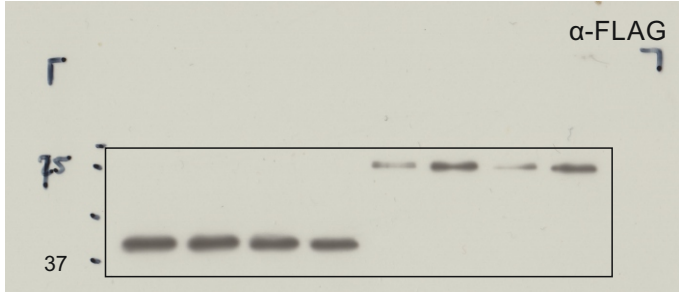

S1. B

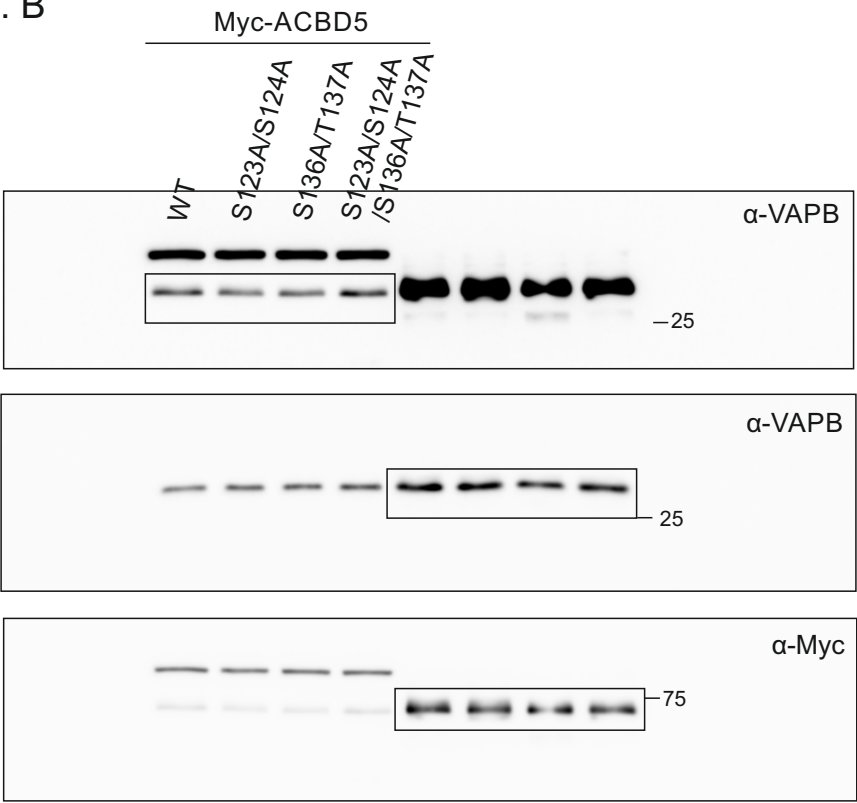

Supplement: SourceData FS1 — contains original blots for Fig. S1. [file JCB_202003143_SourceDataFS1.pdf]

S3. B

FLAG-ACBD4

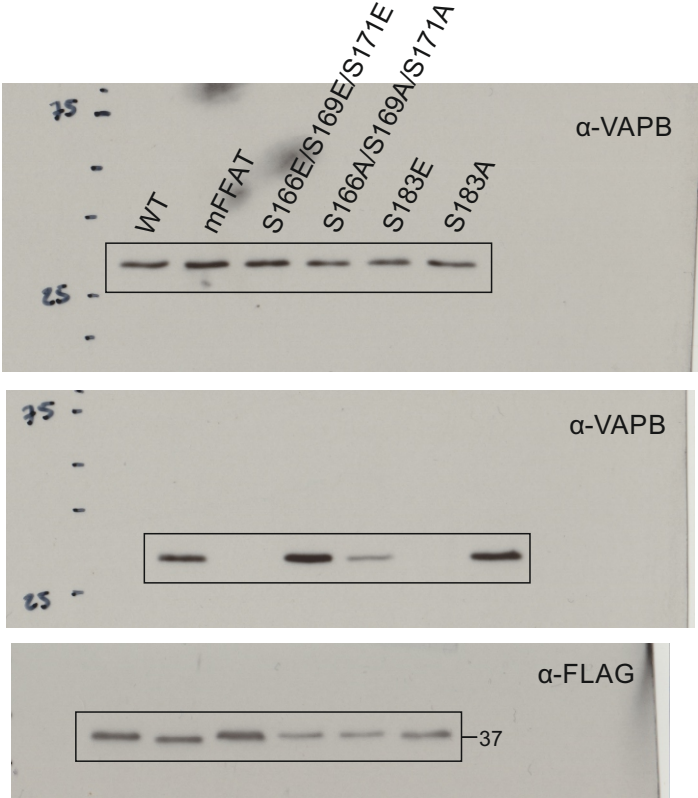

FLAG-ACBD4

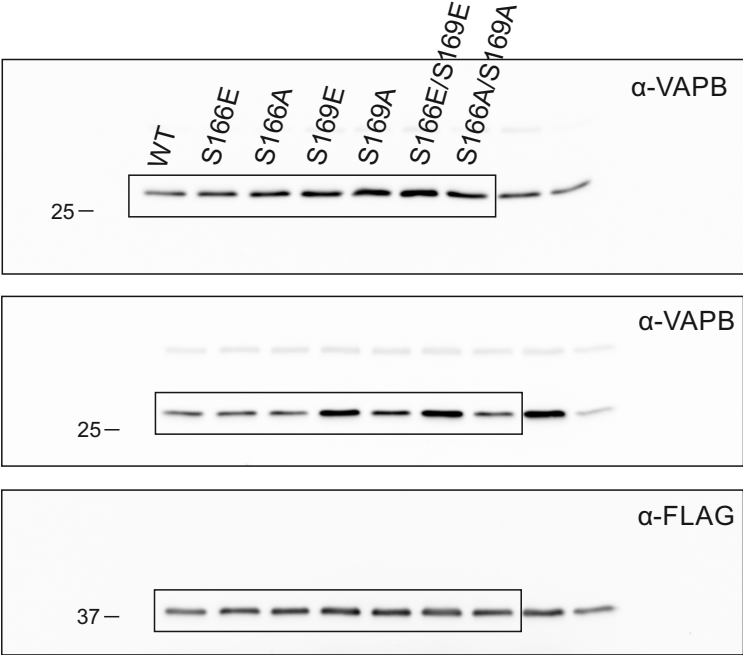

Supplement: SourceData FS3 — contains original blots for Fig. S3. [file JCB_202003143_SourceDataFS3.pdf]

# S4. A

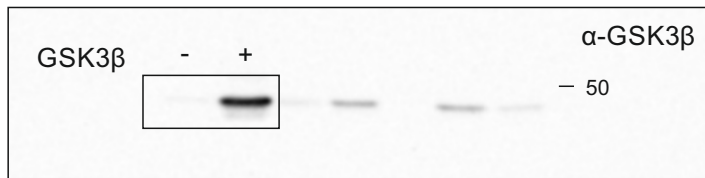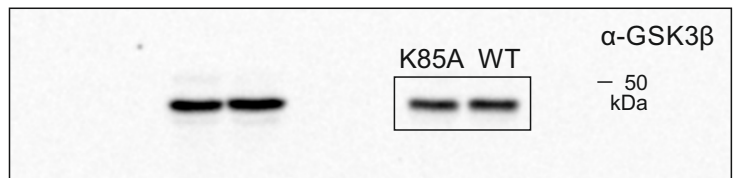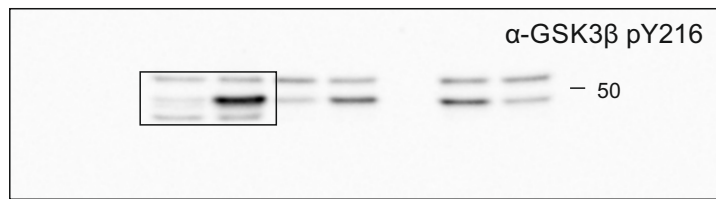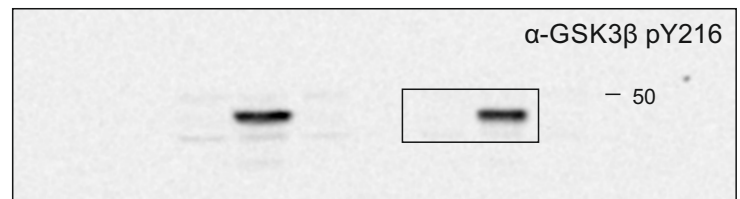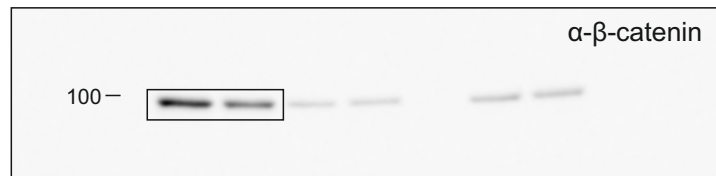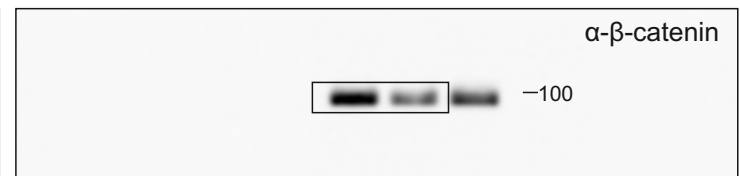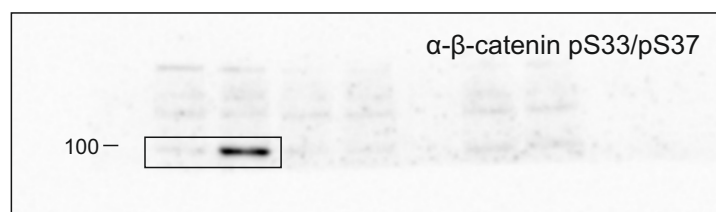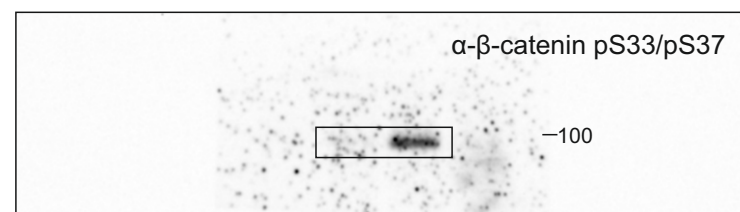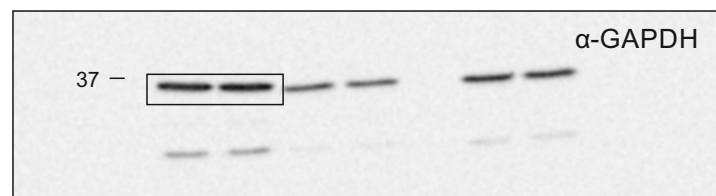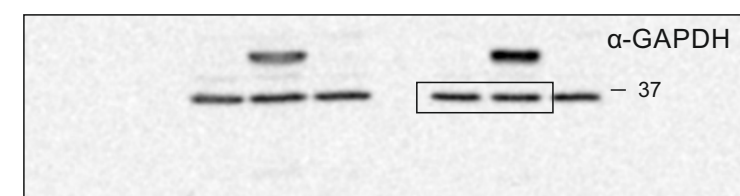

S4. B

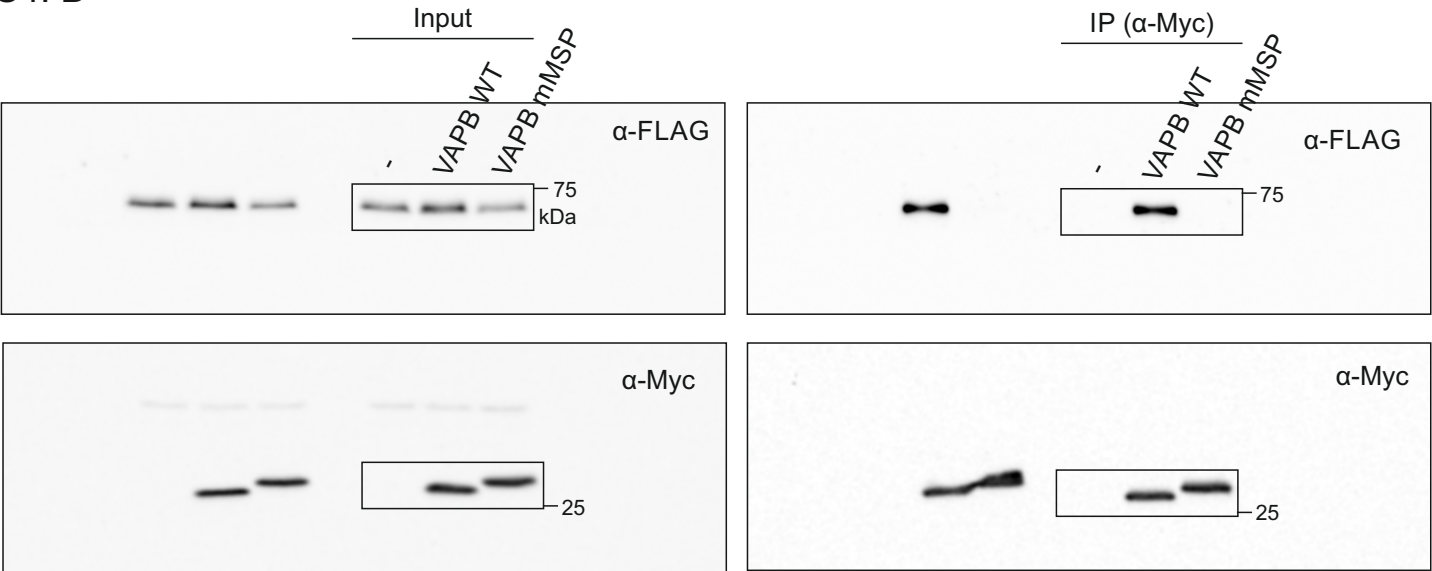

Supplement: SourceData FS4 — contains original blots for Fig. S4. [file JCB_202003143_SourceDataFS4.pdf]
